# The E3 ubiquitin ligase and RNA-binding protein ZNF598 orchestrates ribosome quality control of premature polyadenylated mRNAs

**DOI:** 10.1038/ncomms16056

**Published:** 2017-07-07

**Authors:** Aitor Garzia, Seyed Mehdi Jafarnejad, Cindy Meyer, Clément Chapat, Tasos Gogakos, Pavel Morozov, Mehdi Amiri, Maayan Shapiro, Henrik Molina, Thomas Tuschl, Nahum Sonenberg

**Affiliations:** 1 Howard Hughes Medical Institute and Laboratory for RNA Molecular Biology, The Rockefeller University, 1230 York Ave, Box 186, New York, New York 10065, USA; 2 Department of Biochemistry and Goodman Cancer Research Centre, McGill University, Montreal, Quebec H3A 1A3, Canada; 3 Proteomics Resource Center, The Rockefeller University, New York, New York 10065, USA

## Abstract

Cryptic polyadenylation within coding sequences (CDS) triggers ribosome-associated quality control (RQC), followed by degradation of the aberrant mRNA and polypeptide, ribosome disassembly and recycling. Although ribosomal subunit dissociation and nascent peptide degradation are well-understood, the molecular sensors of aberrant mRNAs and their mechanism of action remain unknown. We studied the Zinc Finger Protein 598 (ZNF598) using PAR-CLIP and revealed that it cross-links to tRNAs, mRNAs and rRNAs, thereby placing the protein on translating ribosomes. Cross-linked reads originating from AAA-decoding tRNA^Lys^(UUU) were 10-fold enriched over its cellular abundance, and poly-lysine encoded by poly(AAA) induced RQC in a ZNF598-dependent manner. Encounter with translated polyA segments by ZNF598 triggered ubiquitination of several ribosomal proteins, requiring the E2 ubiquitin ligase UBE2D3 to initiate RQC. Considering that human CDS are devoid of >4 consecutive AAA codons, sensing of prematurely placed polyA tails by a specialized RNA-binding protein is a novel nucleic-acid-based surveillance mechanism of RQC.

## Abstract (web summary)

Translation of aberrant mRNAs causes ribosome stalling and translation arrest, followed by recycling of the stalled ribosome complex. Here the authors show that the Zinc Finger Protein 598 (ZNF598/Hel2) is implicated in sensing faulty translation of prematurely polyadenylated mRNAs through the recognition of AAA codons.

Cryptic polyadenylation within coding sequences (CDS) or incompletely removed introns produce aberrant transcripts that lack in-frame stop codons^1^. Translation of such mRNAs may result in proteins prone to malfunction and deleterious effects on cells^2^,^3^,^4^. To mitigate these errors, cells have developed quality-control processes to monitor translating mRNAs and detect aberrant mRNAs, such as those with premature polyA tails within their CDS. Defects in components of the surveillance machineries have been implicated in several types of diseases including neurodegeneration and cancer^5^,^6^.

The ribosome-associated quality control (RQC) is a mechanism that senses the state of mRNA translation and detects ribosome stalling at the site of defective mRNAs, which results in targeting of both the translating mRNA and nascent peptide for degradation^7^. RQC can be divided into several steps, surveillance of the translating mRNA and detection of stalled ribosome, ribosomal subunit dissociation, and degradation of the defective mRNA and nascent peptide. Although the processes of ribosomal subunit dissociation and nascent peptide degradation are well studied^8^,^9^,^10^,^11^,^12^, the mechanism of surveillance of the translating mRNA and detection of stalled ribosome, in particular the molecular sensors of aberrant mRNAs and their mechanism of action, remain largely unknown. Earlier studies suggested that presence of the polyA sequences within the CDS causes ribosome stalling through interactions between the positively charged peptide (poly-lysine) and the negatively charged exit channel of the ribosome^8^,^13^,^14^. However, others showed that at least in mammalian cells RQC at poly-lysine sites is codon-sequence dependent as runs of poly-lysine residues coded by AAA codons induced ribosome stalling much more efficiently than equivalent runs of poly-lysine encoded by AAG codons^15^. These results indicate that sensing A-rich mRNA sequence in mammalian cells dominates over general polybasic amino-acid-triggered translational regulation. Nevertheless, the mechanisms by which premature polyA sequences are detected in aberrant mRNAs and the following molecular events leading to ribosome stalling are not known.

In yeast, the E3 ubiquitin ligase Hel2 has been implicated in facilitating the earlier steps of RQC at polybasic sequences^8^. Notably, Hel2-dependent K63 polyubiquitination is necessary for the initial processes involved in stalled translation surveillance^16^. However, the precise functions of Hel2 in detection of stalled ribosomes or its ubiquitination substrates have not been identified. The Zinc Finger Protein 598 (ZNF598) is the human ortholog of Hel2 and contains a RING domain characteristic of E3 ubiquitin ligases and several C2H2-type zinc finger motifs, commonly found in nucleic acid-binding proteins^17^,^18^. We previously described ZNF598 protein in a complex with the translation repressor proteins EIF4E2/4EHP and GIGYF2 (ref. 19). Two recent reports showed that ZNF598 is also required for stalling at polyA sequences and linked its E3 ubiquitin ligase activity to translation arrest through ubiquitinating the 40S subunit ribosomal proteins RPS10 and RPS20 (refs 20, 21).

Here, we reveal that ZNF598 directly binds to the translating mRNA and tRNAs on ribosomes and triggers ribosome stalling and RQC at premature polyA sequences. We further identified RPS3A as an additional substrate of ZNF598 E3 ubiquitin ligase activity, and UBE2D3 as the ZNF598-interacting E2 ubiquitin ligase. Our findings establish a link between the RNA-binding properties and ubiquitin ligase activity of a uniquely conserved protein in monitoring mRNA translation.

## Results

### ZNF598 associates with translating ribosomes

**Figure 1:**
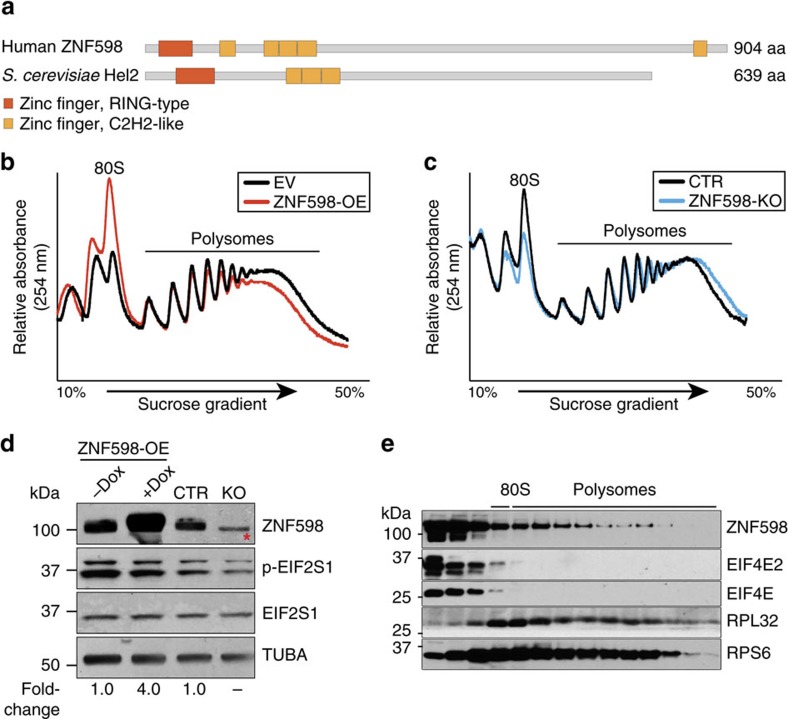
ZNF598 is a translation repressor that sediments with polysomes. (**a**) Domain organization of the human ZNF598 protein and its yeast orthologue Hel2 as determined by Interpro. The protein length in amino acids (aa) is indicated. (**b**) Polysome profiles of empty vector (EV) and ZNF598-OE HEK293 cells. (**c**) Polysome profiles of parental (CTR) and ZNF598-KO HEK293 cells. (**d**) Western blot analysis of ZNF598 expression and EIF2S1 phosphorylation (p-EIF2S1) to probe for proteotoxic stress in ZNF598-OE and ZNF598-KO cells and controls. * Indicates a non-specific band recognized by the anti-ZNF598 antibody (GeneTex). Numbers indicate the ratio of ZNF598 expression relative to CTR cells. (**e**) Western blot analysis with the indicated antibodies of fractions of the ZNF598-OE HEK293 cell lysates after separation over a 10–50% sucrose gradient. The position of 80S ribosomes and polysomes in the gradient is indicated.

Human ZNF598 encodes a ubiquitously expressed 904 amino acid (aa) protein containing one *N*-terminal RING domain characteristic of E3 ubiquitin ligases and four *N*-terminal and one *C*-terminal C2H2-type zinc finger motifs (Fig. 1a & Supplementary Fig. 1). To evaluate its potential role in translational control, we performed polysome profiling using ZNF598 overexpression (ZNF598-OE) or ZNF598 knockout HEK293 cells (ZNF598-KO; Supplementary Fig. 2). ZNF598-OE induced a shift from heavy polysomes to monosomes and ZNF598-KO induced a shift to heavier polysomes, indicating translational repression (Fig. 1b,c). This effect was independent of EIF4E2 (Supplementary Fig. 3) and was neither due to general translational repression mediated by phosphorylation of eukaryotic initiation factor 2α EIF2S1/eIF2α (Fig. 1d) under stress condition. Western blot analysis of the polysome fractions showed that a proportion of ZNF598 protein was associated with heavy polysomes in ZNF598-OE cells (Fig. 1e). Size exclusion chromatography also revealed that ZNF598 protein co-fractionated with ribosomes in a ≥2 MDa complex (Supplementary Fig. 4), suggesting that ZNF598 either interacted transiently with assembled ribosomes or associated with a subset of actively translating ribosomes. Immunofluorescence analysis of ZNF598 upon exposure to arsenite-induced stress did not reveal any change in its cytosolic distribution, unlike many known cytosolic RNA-binding proteins or 18S ribosomal RNA which accumulate in stress granules^22^,^23^ (Supplementary Fig. 5). Together, these observations support a role of ZNF598 in translation.

### ZNF598 binds to RNAs associated with translating ribosomes

**Figure 2:**
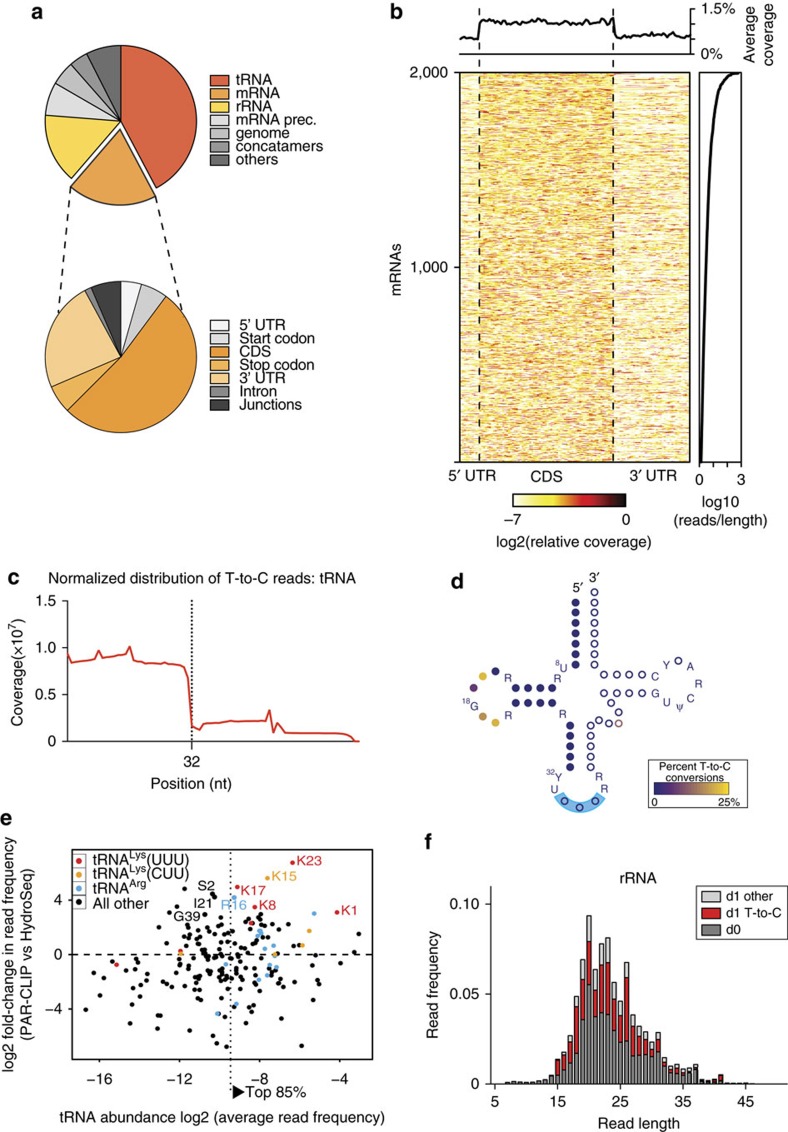
PAR-CLIP RNA targets of ZNF598 in HEK293 cells. (**a**) Relative composition of ZNF598 PAR-CLIP sequence reads mapping to each RNA category with up to two mismatches. The reads mapped to nuclear encoded mRNAs are further subdivided into functional regions. (**b**) Meta-gene plot of PAR-CLIP reads mapping to mRNA defined by at least one read with T-to-C conversion. Each row in the matrix represents the relative coverage over each mRNA. mRNAs are ranked by the number of mapped T-to-C reads for the 3,000 most abundant mRNAs. The upper panel depicts the average coverage over the top 3,000 mRNAs. (**c**) Bin-normalized distribution of ZNF598 PAR-CLIP T-to-C reads mapping to tRNAs. (**d**) Schematic diagram of the secondary structure of tRNAs. Conserved nucleotides across cytosolic tRNAs are spelled out in letters, while non-conserved nucleotides are depicted by circles. The colour-code indicates the T-to-C conversion ratio. Filled circles at the 5′ end represent nucleotides covered by ZNF598 PAR-CLIP sequence reads (32 nt of the 5′ end). (**e**) Relative changes in tRNA abundance in ZNF598 PAR-CLIP versus HydroSeq (total cellular tRNA). All tRNA^Lys^(UUU) sequence variants are coloured in red, tRNA^Lys^(CUU) variants are coloured in orange, and all tRNA^Arg^ variants are coloured in blue. tRNAs, which are over-represented in PAR-CLIP with a false discovery rate (FDR) of <5% are labelled by their corresponding gene names. tRNAs collecting the top 85% of sequencing reads are to the right and residual tRNAs are to the left of the dotted vertical line. (**f**) Average read composition of two replicates of ZNF598 PAR-CLIP experiments for the rRNA category. Reads were assigned as d0 (dark grey), d1 T-to-C (red), d1 other than T-to-C and (light grey).

To investigate ZNF598 function and identify its target RNAs, we performed 4-thiouridine (4SU) photoactivatable ribonucleoside-enhanced cross-linking and immunoprecipitation (PAR-CLIP)^24^ in ZNF598-OE HEK293 cells (Supplementary Figs. 2a and 6a). We observed that ZNF598 cross-linked to tRNAs, mRNAs and rRNAs (Fig. 2a, Supplementary Fig. 6b–g & Supplementary Data 1). The average ratio of cross-linked reads annotated as tRNAs, mRNAs and rRNAs was ∼4:2:1. Cross-linked reads derived from mRNAs showed evenly distributed enrichment for CDS over untranslated regions (UTRs) (Fig. 2a,b). The cross-linked mRNA read abundance resembled the overall mRNA abundance in HEK293 cells as determined by polyA mRNA-Seq (Supplementary Fig. 7). The reads mapped to nuclear encoded cytoplasmic tRNAs originated predominantly from their 5′ halves, which were cross-linked to ZNF598 via their D-loops (Fig. 2c,d). Although ZNF598 protein cross-linked to every cytoplasmic tRNA, cross-linked reads unique to tRNA^Lys^(UUU) were ∼10-fold enriched relative to their total cellular abundance, whereas cross-linked reads to tRNA^Lys^(CUU) only displayed an average twofold enrichment (Fig. 2e & Supplementary Data 2). Cross-linked reads to rRNA (Fig. 2f) originated predominantly from 5S (pos. 96–121) and 18S rRNAs (pos. 686–707 and 745–778) and to a lesser extent from 5.8S and 28S (Supplementary Figs. 8 and 9). Taken together, the cross-linked RNA targets and positional cross-linking spectra indicate that a fraction of ZNF598 protein was bound to translating ribosomes. In light of the enrichment of cross-linking to (AAA)-decoding tRNA^Lys^(UUU), we hypothesized that ZNF598 may have a role in detecting premature polyA tails or protein-folding problems of poly-lysine rich proteins that would induce ribosome stalling and RQC pathway^7^,^13^. The transcript abundance of ZNF598 is ∼10-fold below the average of transcripts encoding ribosomal proteins but similar in abundance to other proteins implicated in RQC (Supplementary Data 3). Considering that direct molecular contacts are required for photo-cross-linking of ZNF598 to the CDS of mRNAs, tRNAs and rRNAs, we conclude that ZNF598 protein is associated with translating ribosomes and intimately monitors the CDS of translated mRNAs and/or identity of tRNAs occupying the ribosome and triggering RQC upon encounter with premature polyA tails.

### ZNF598 initiates RQC at premature polyA sequences

**Figure 3:**
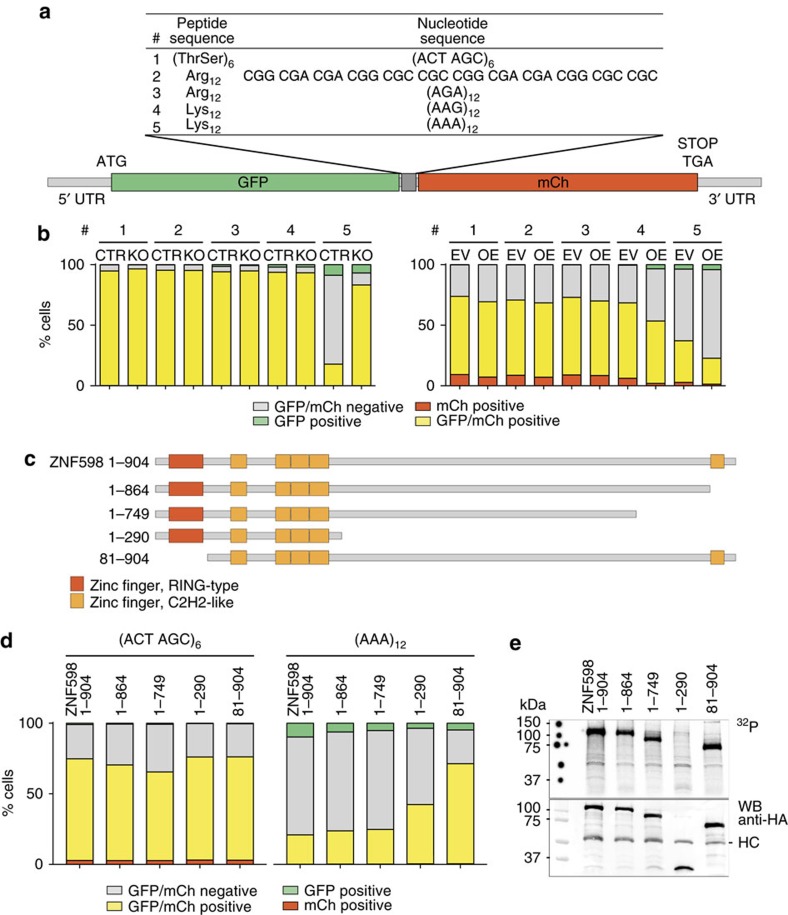
The RING domain of ZNF598 is essential for ribosome stalling at polyA residing within coding sequences. (**a**) Schematic diagram of the reporter constructs sandwiching a polybasic oligopeptide track between the fluorescent GFP and mCherry (mCh) fusion protein. (ACT AGC)_6_ [(ThrSer)_6_] encoded a neutrally charged amino-acid tract that served as a control. (**b**) Detection of GFP and mCherry fluorescent signals by FACS analyses in samples from (**a**) shown as relative cell numbers. Each experiment was performed in triplicates. (**c**) Domain structures of ZNF598 full-length and truncation mutants, with numbers referring to the position of amino acids. (**d**) Detection of GFP and mCherry fluorescent signals by FACS analyses in samples expressing full-length or truncated versions of ZNF598 and transiently transfected with GFP-mCherry reporter with (ACT AGC)_6_ and (AAA)_12_ linkers. Each experiment was performed in triplicate. (**e**) Upper panel: autoradiograph of cross-linked, ^32^P-labelled, RNA- Flag/HA-ZNF598 immunoprecipitate. Flag/HA-tagged full-length ZNF598 or truncated versions were separated by SDS-PAGE after 4SU PAR-CLIP. Lower panel: Anti-HA Western blot analyses of the cross-linked RNA-protein immunoprecipitates; HC, antibody heavy chain.

In yeast, Hel2 facilitates ribosome stalling at both poly-lysine and poly-arginine polybasic amino-acid coding sites^8^,^16^,^25^,^26^. Polybasic peptides are lysine- (AAA or AAG codons) and/or arginine-rich (CGU, CGC, CGA, CGG, AGG or AGA codons). To determine whether the amino acid or the mRNA sequence is responsible for ribosome stalling, we generated stable HEK293 reporter cell lines for parental (CTR) and ZNF598-KO expressing GFP and mCherry fusion proteins separated by a 12 aa spacer composed of various lysine, arginine or control threonine-serine codon repeats (Fig. 3a). We observed fourfold decreased expression of the fusion protein separated by a (AAA)_12_ poly-lysine-coding sequence as compared with either control poly-threonine-serine-coding (ACT AGC)_6_, poly-lysine-coding (AAG)_12_, poly-arginine (AGA)_12_, or poly-arginine-coding combinations of CGC, CGA and CGG. Importantly, expression of the (AAA)_12_ reporter reverted to the level of the (ACT AGC)_6_ control in ZNF598-KO cells (Fig. 3b & Supplementary Fig. 10b left panels), demonstrating ZNF598-dependent repression of the (AAA)_12_ reporter. The expression of the reporter fusion proteins was also studied in ZNF598-OE and its empty vector control (EV) using transient reporter plasmid transfection, revealing further decreased (AAA)_12_ reporter expression as compared with the (ACT AGC)_6_ control (Fig. 3b & Supplementary Fig. 10b right panels). The increased repression of the (AAA)_12_ reporter in ZNF598-OE cells also suggests that ZNF598 protein is sub-stoichiometric to ribosomes at standard conditions. Translational repression of the (AAA)_12_ reporter was not associated with an imbalance of the GFP to mCherry signal ratio (Fig. 3b) or accumulation of truncated fusion protein (Supplementary Fig. 10b), which is consistent with activation of the RQC pathway and destruction of the nascent reporter GFP segment^8^. These results demonstrate that whereas both poly-lysine and poly-arginine sequences induce ribosome stalling and RQC in yeast^8^,^16^,^26^, only poly-lysine, encoded by the AAA codon induces RQC in mammalian cells in a ZNF598-dependent manner. Therefore, sensing A-rich mRNA sequence in mammalian cells dominates over general polybasic amino-acid-triggered translational regulation, as reported previously^15^.

Interestingly, although there are 7,433 (AAA)_2_ and 175 (AAA)_3_ consecutive codons in human CDS, multiple consecutive (AAA) codons are scarce and do not exceed more than four (Supplementary Fig. 11 and Supplementary Data 4). This implies that ZNF598 protein may be directly involved in detecting aberrant CDS containing ≥12 nt polyA during translation, and/or senses the simultaneous occupation of the ribosomal A, P and E sites with tRNA^Lys^(UUU), and/or a translating ribosome conformational transition as a consequence of the above. The multiple C2H2-type zinc fingers positioned along the protein may be implicated in such function.

### The *N*-terminal RING domain is critical for ZNF598 function

To identify the protein domains of ZNF598 critical for triggering premature polyA-dependent ribosome stalling and RQC, we complemented the ZNF598-KO cells with full-length or a series of truncated mutants of ZNF598 (Fig. 3c). Although the *C*-terminal C2H2 domain (aa 864–904) was dispensable for RQC of the reporter without compromising RNA cross-linking, the *N*-terminal RING domain (lacking in the 81–904 truncation) was required for RQC (Fig. 3d), albeit RNA cross-linking and thus translating ribosome binding was unaffected by deletion of the RING domain (Fig. 3e & Supplementary Fig. 12). These functional observations were corroborated by polysome profiling, which show the requirement of the RING domain and unstructured central domain, but not the *C*-terminal C2H2 motif for translational repression of the endogenous mRNAs (Supplementary Fig. 13).

### Ubiquitination of ribosomal proteins is required for RQC

**Figure 4:**
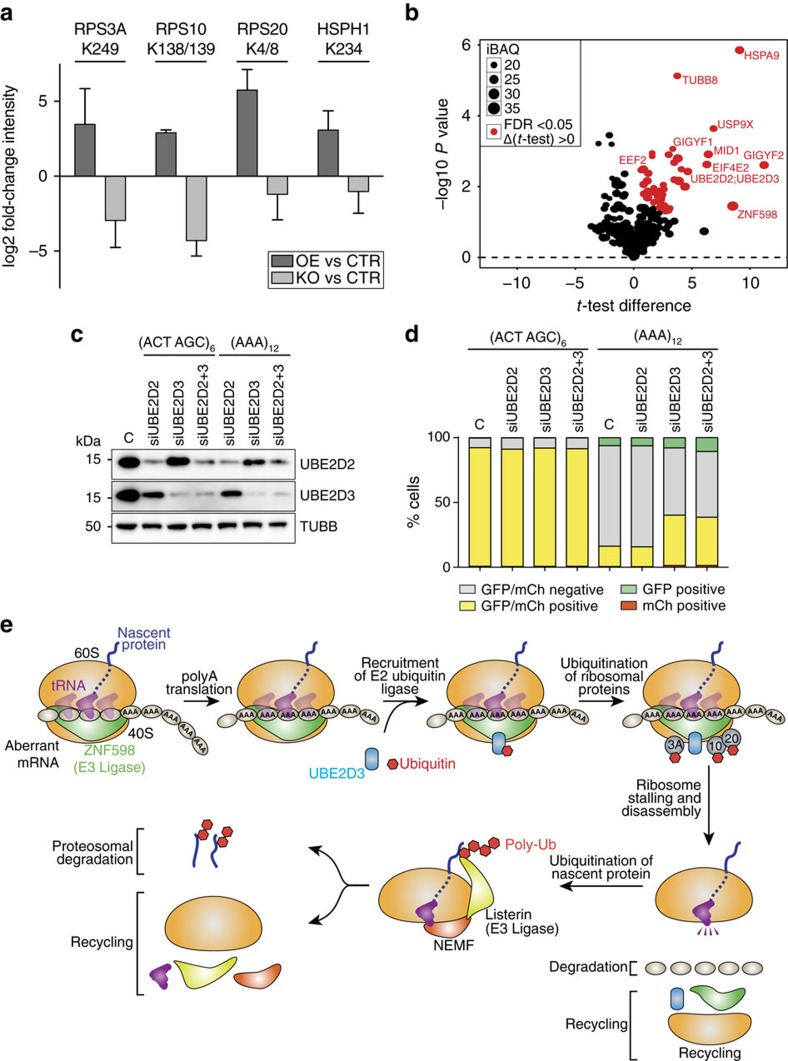
Ribosome stalling at coding polyA sequences requires the E3 ubiquitin ligase activity of ZNF598 and the E2 ubiquitin ligase UBE2D3. (**a**) Identification of differentially ubiquitinated proteins by ubiquitin remnant immuno-affinity profiling. Log2 ratios of the enrichment of the quantified diGly-containing peptides are shown. Only peptides with an average log2 ≥2 upregulation for ZNF598OE/CTR and downregulation for ZNF598KO/CTR are shown. Error bars represent the s.d. (*n*=2). See Supplementary Data 5 for a complete list of all detected peptides. (**b**) Volcano plots of the quantitative proteomic analysis of the ZNF598 interactome. The *t*-test difference based on label free quantitation for each detected protein is plotted against the negative logarithmic *P* value of a Welch’s *t*-test. The intensity based absolute quantitation (iBAQ)^51^ values correspond to the sum of all the peptide intensities divided by the number of observable peptides of a protein and are represented by point size. Proteins with a permutation-based FDR-value of <5% and *t*-test difference >0 are labelled in red and represent putative ZNF598 interactors (see also Supplementary Fig. 15 and Supplementary Data 6). (**c**) Analysis of siRNA-mediated knockdown of UBE2D2, UBE2D3 or both UBE2D2 and UBE2D3 in HEK293 cells by western blot. C indicates mock transfection. (**d**) Detection of GFP and mCherry fluorescent signals by FACS analyses in samples from (**c**), for reporter constructs containing (ACT AGC)_6_ and (AAA)_12_ linkers. Each experiment was performed in triplicate. (**e**) Model for ZNF598-dependent ribosome stalling and RQC at cryptic polyadenylated protein-coding mRNAs.

RING finger proteins coordinate the transfer of ubiquitin to substrate proteins by the recruitment of E2 ubiquitin ligases^27^. Although Hel2-dependent ubiquitination has been implicated in the initial surveillance process of stalled ribosomes in yeast^16^, the target substrates of Hel2 or its corresponding E2 ubiquitin ligase were not known. The *N*-terminal domains of Hel2, including the RING domain, are the most conserved in ZNF598 (Supplementary Fig. 1), suggesting a similar E3 ligase activity for ZNF598. To identify proteins targeted by ZNF598-dependent ubiquitination, we used ubiquitin remnant immuno-affinity profiling^28^ in control, ZNF598-OE and ZNF598-KO HEK293 cells. Three small ribosomal subunit proteins RPS3A (eS1), RPS10 (eS10) and RPS20 (uS10), and the heat-shock protein HSPH1 were enriched more than fourfold in ZNF598-OE cells and decreased more than fourfold in ZNF598-KO cells as compared with CTR cells (Fig. 4a and Supplementary Data 5). In addition to priming the target proteins to proteasome degradation, ubiquitination also has a regulatory function in diverse molecular pathways such as DNA repair^29^, anti-viral immunity^30^ and signal transduction^31^. Evolutionarily conserved, regulatory ubiquitination of the small ribosomal subunit proteins induced by inhibitors of translation elongation has previously been reported in mammalian cells^32^. Western blot analysis revealed that overexpression or depletion of ZNF598 failed to affect the expression of RPS3A, RPS10 and RPS20 proteins (Supplementary Fig. 14), indicating that the ZNF598-regulated ubiquitination of small ribosomal subunit proteins is likely a signalling event rather than inducing protein degradation by the proteasome.

Further biochemical experiments were performed to investigate the functional consequence of ZNF598-mediated ubiquitination of RPS3A, RPS10 and RPS20. We generated stable cell lines expressing the *C*-terminus Myc-DDK-tagged wild-type or mutant RPS3A, RPS20 or RPS10 in which lysines that are subject to ubiquitination were substituted by arginine. For RPS10 and RPS20, where multiple lysines were ubiquitinated (RPS10; K138, K139 and RPS20; K4, K8), we also created double mutants. Western blot analysis confirmed that the mutant proteins were at least as abundantly expressed as the wild-type proteins (Supplementary Fig. 15a–c). However, only RPS10 K138R, and the double mutant K138R/K139R, partially impaired RQC for the (AAA)_12_ reporter, whereas mutations in RPS20 and RPS3A failed to effect (AAA)_12_ reporter expression (Supplementary Fig. 15d). This indicates that downstream ribosomal protein ubiquitination of RPS10 contributes to RQC.

Recent reports also identified ZNF598-dependent ubiquitination of ribosomal proteins RPS10 and RPS20 as ZNF598-stimulated ubiquitin conjugates; although these studies differ with respect to the relative importance of the two proteins as well as their ubiquitination sites^20^,^21^. Both studies showed the requirement for ubiquitination of RPS10 in polyA-induced ribosome stalling. Nevertheless, while Juszkiewicz and Hegde^21^ observed only a partial effect in RPS20 K4R/K8R double mutant, Sundaramoorthy, *et al*.^20^ observed that both RPS20 and RPS10 mutants displayed enhanced readthrough of the polyA sequence. We detected RPS3A as an additional target of ZNF598-regulated ubiquitination, which is surprising considering its distance from RPS10 and RPS20 in the ribosome^33^,^34^. However, as mutations in RPS3A did not impact the polyA-induced ribosome stalling, it is likely that ubiquitination of this protein occurs or has a role in downstream events such as during ribosomal subunit dissociation.

RING family E3 ubiquitin ligases catalyse the transfer of ubiquitin from an E2 enzyme to target proteins^35^. To identify the E2 ligase for ZNF598 we performed immunoprecipitation (IP) in ZNF598-OE HEK293 cells followed by mass spectrometry (IP/MS) analysis of the immunoprecipitate. We identified the 97% identical UBE2D2 and UBE2D3 E2 ligases among the most significant hits (Fig. 4b, Supplementary Figs. 16 and 17a & Supplementary Data 6). Their interactions with ZNF598 were verified by IP and western blot analysis, which revealed that UBE2D3 (the most abundant homologue in HEK293 cells; Supplementary Fig. 17b), but not UBE2D2, specifically interacted with ZNF598 (Supplementary Fig. 17c,d). UBE2D3 also co-fractionated with ZNF598 and ribosomal proteins in ≥2 MDa complexes (Supplementary Fig. 18), but failed to co-fractionate in heavy complexes with the ΔRING mutant (aa 81–904) of ZNF598 (Supplementary Fig. 12). In addition, knockdown of UBE2D3, but not UBE2D2, partially abrogated RQC on the (AAA)_12_ reporter (Fig. 4c,d) or translational repression of endogenous mRNAs in ZNF598-OE cells (Supplementary Fig. 19). These results underscore the requirement of the E3 ligase activity of ZNF598 and highlight the regulatory ubiquitination of the ribosomal proteins RPS3A, RPS10 and RPS20 in ribosome stalling and RQC.

Although ZNF598 was originally identified as a component of the ZNF598/GIGYF2/EIF4E2 complex, we showed that ZNF598-dependent translational repression was independent of the cap-binding EIF4E2 protein. Considering that EIF4E2 represses mRNA translation^19^, we propose that this complex may have a role in downstream degradation of premature polyadenylated mRNAs via displacement of the canonical cap-binding protein EIF4E by the repressive EIF4E2 homolog. The sub-stoichiometric abundance of ZNF598 as well as other proteins implicated in RQC sensing deleterious amino-acid repeats, premature stop codons or truncated non-polyadenylated mRNAs, suggest that subpopulations of error-sensing ribosomes distributed randomly among translating polysomes divide the labour of detecting faulty mRNAs. The study of molecular and functional ribosome heterogeneity^36^ will provide further direction in elucidating mechanisms of RQC. Although the human genome encodes 864 proteins with RING domain and 2,474 proteins containing at least one C2H2 domain, only three additional proteins, TRIM23, ZNF645 and CBLL1, carry a combination of RING and C2H2 domains (http://www.ebi.ac.uk/interpro/protein/), but none is as strongly evolutionarily conserved as ZNF598 (Supplementary Fig. 20). In as much these proteins contribute to other possibly less conserved ubiquitination-dependent RNA pathways outside of translation, it poses an intriguing question. In summary, we showed that ribosome stalling and RQC in mammalian cells at premature polyA-containing mRNAs involved recognition of the tRNA^Lys^(UUU) and/or the mRNA (AAA) codon repeats by the unique RNA-binding and E3 ligase protein ZNF598 (see model; Fig. 4e).

## Methods

### Cell lines and culture conditions

Flp-In T-REx 293 cells (Thermo Fisher Scientific, R78007) were grown in high glucose Dulbecco's Modified Eagle's Medium (DMEM) (Thermo Fisher Scientific, 11965118) supplemented with 10% v/v fetal bovine serum (FBS), 100 U ml^−1^ penicillin, 100 μg ml^−1^ streptomycin, 2 mM L-glutamine, 100 μg ml^−1^ zeocin and 15 μg ml^−1^ blasticidin. Presence of mycoplasma contamination in cells was tested by mRNA-Seq. Cell lines inducibly expressing 3xFlag-ZNF598 or Flag/HA-tagged truncated ZNF598 variants were generated as described previously^37^ and selected and maintained in media supplemented with 100 μg ml^−1^ hygromycin. Expression of tagged proteins was induced for 24 h by the addition of doxycycline at 1 μg ml^−1^ final concentration. In the text, figures and tables, parental Flp-In T-REx 293 cells are labelled as CTR and the CRISPR-Cas9-mediated knockout as ZNF598-KO. 3xFlag-ZNF598 cells are labelled as ZNF598-OE. Because they were grown in different media than the CTR and ZNF598-KO cells, ZNF598-OE cells were always compared with control cells expressing 3xFlag EV, labelled as EV. For experiments with truncated variants, cells expressing full-length or truncated variants of ZNF598 were established using ZNF598-KO cells.

### Antibodies and RNA interferences

The following antibodies were used: mouse anti-β-tubulin (Sigma, T4026; 1:5,000 dilution), mouse anti-α-tubulin (Santa Cruz, sc-23948; 1:1,000 dilution), mouse anti-β-actin (Sigma, A5441; 1:1,000 dilution), mouse anti-Flag M2 (Sigma, F3165; 1:2,000 dilution), mouse anti-RPS6 (Cell Signaling, C-8; 1:2,000 dilution), rabbit anti-RPS3A (Abcam, ab171742; 1: 2,000 dilution), rabbit anti-RPS10 (Abcam, ab151550; 1: 2,000 dilution), rabbit anti-RPS20 (Abcam, ab133776; 1: 2,000 dilution), rabbit anti-Rack1 (Cell Signaling, 4716; 1:2,000 dilution), rabbit anti-EEF2 (Cell Signaling, 2332; 1:1,000 dilution), rabbit anti-4EHP (GeneTex, GTX103977; 1:500 dilution), mouse anti-HA (Fisher; 50-103-0108; 1:2,000 dilution), rabbit anti-UBE2D2 (Abcam, ab155088; 1:2,000 dilution), mouse anti-UBE2D3 (Abcam, ab58251; 1:2,000 dilution), mouse anti-GFP (Clontech, 632375; 1:1,000 dilution), rabbit anti-ZNF598 (Abcam, ab135921; 1:500 dilution), rabbit anti-ZNF598 (a gift from Jianxin Xie at Cell Signaling Technology; 1:1,000 dilution), rabbit anti-ZNF598 (GeneTex, GTX119245; 1:500 dilution). For ZNF598 the Cell Signaling antibody was used in most experiments, unless stated otherwise. Uncropped images of all of the immune-blots in this manuscript are shown in Supplementary Figs 21 and 22.

The following siRNA and shRNAs were used: ON-TARGETplus Non-targeting Control Pool (Dharmacon, D-001810-10-05), UBE2D2 siRNA SMARTpool (Dharmacon, L-010383-00-0005), UBE2D3 siRNA SMARTpool (Dharmacon, L-008478-00-0005), Non-Targeting shRNA Controls (Sigma, SHC002) and EIF4E2 shRNA (Sigma, TRCN0000152006).

### Lentivirus production

A total of 8 × 10^6^ 293FT (Thermo Fisher Scientific, R70007) cells were cultured in a 10-cm dish for 24 h in high-glucose DMEM supplemented with 10% v/v FBS. Medium was replaced by OptiMEM (Thermo Fisher Scientific, 51985091) 30 min before transfection. Lentivirus particles were produced by transfecting the 293FT cells using Lipofectamine 2000 (Thermo Fisher Scientific, 11668019) and 10 μg shRNA plasmid, 6.5 μg psPAX2 (Addgene, plasmid 12260) and 3.5 μg pMD2.G (Addgene, plasmid 12259) packaging plasmids. 5 h post transfection, the medium was replaced with fresh high-glucose DMEM supplemented with 10% v/v FBS. Supernatant was collected at 48 h post transfection, replaced with fresh medium and harvested again after 24 h. Viral particles were cleared by filtration (45 μm; Fisher Scientific, 09-720-005) and virus titre was measured by colony formation assay using 293FT cells. The multiplicity of infection was adjusted to ∼5. Virus solution was stored at −80 °C without cryopreservative in 1 ml aliquots or used to infect the cells directly in the presence of 6 μg ml^−1^ polybrene (Sigma, H9268).

### Generation of ZNF598 knockout cell lines

CRISPR-Cas9-mediated genome editing was performed as described previously^38^. We designed three small guide RNAs (sgRNAs) cognate to the coding region of ZNF598 gene: 5′-CTACTGCGCCGTGTGCCGCG, 5′-GAAAGGTGTACGCATTGTAC, and 5′-TACGCATTGTACAGGTGAGC. The following primers were used for the PCR-genotyping: sense primer1, 5′-GGAGGCGGAGGCGGCGGCAGC; anti-sense primer1, 5′-CCCCGCCCTGGGTGGCCCCACC; sense primer2, 5′- GGGGGTCCCATCCCAGTCCTGC; anti-sense primer2, 5′- CCTGGCCCCAGCATTGGTGCACC. PCR products were cloned using the Zero Blunt PCR Cloning Kit (Thermo Fisher Scientific, K270040) and 11 clones were sequenced per cell line to verify successful genome editing.

### Plasmid construction

For overexpression, ZNF598 cDNA was cloned into pcDNA5/FRT/TO/3xFlag/N-term plasmid (a gift from Dr Jernej Ule) by digestion with *Kpn*I and *Not*I and subsequent use of T4 DNA ligase. pENTR4 (Thermo Fisher Scientific, A10465) plasmids for expression of ZNF598 full-length or truncated variants were generated by PCR amplification of the respective sequences adding attB1 and attB2 recombination sites to the primers followed by recombination using the Gateway BP recombinase (Thermo Fisher Scientific, 11789020). pENTR4 plasmids were recombined into the pFRT/TO/Flag/HA-DEST destination vector (Thermo Fisher Scientific) using the GATEWAY LR recombinase (Thermo Fisher Scientific, 11791020). Reporters expressing GFP, mCherry, or GFP-mCherry fusion proteins separated by 12 amino-acid spacers were cloned into pcDNA5/FRT (Thermo Fisher Scientific, V6010-20) by digestion with BamHI and NotI and subsequent use of T4 DNA ligase. For expression of ribosomal proteins, wild-type cDNAs were cloned into pLenti-C-Myc-DDK-IRES-Puro (Origene) plasmid by digestion with AscI and MluI. Further mutagenesis was performed using QuikChange Lightning Multi Site-Directed Mutagenesis Kit (Agilent).

### Fluorescence-activated cell-sorting analysis (FACS)

CTR and ZNF598-KO cells with stable, and EV and ZNF598-OE cells with transient, expression of GFP, mCherry and GFP-mCherry fusion proteins separated by 12 amino-acid spacers were sorted and analysed by flow cytometry (LSR II, BD Biosciences).

### Polysome profiling

A total of 20 × 10^6^ cells in a 15-cm plate were pretreated with cycloheximide (100 μg ml^−1^; BioShop Canada, CYC003) for 5 min, collected by centrifugation at 4 °C for 5 min and lysed in 500 μl hypotonic buffer containing 5 mM Tris-HCl, pH 7.5, 2.5 mM MgCl_2_, 1.5 mM KCl, complete EDTA-free protease inhibitor cocktail (Roche, 04693159001), 100 μg ml^−1^ cycloheximide, 2 mM DTT, 200 U ml^−1^ RNasin (Promega, N2111), 0.5% v/w Triton X-100, and 0.5% v/w sodium deoxycholate using 1.5 ml microtubes. The lysates were cleared by centrifugation at 20,000 × g for 5 min at 4 °C. Total RNA concentration in the supernatant was measured by NanoDrop 2000 (Thermo Fisher Scientific) at 254 nm supernatant, and the equivalent of 300 μg of RNA was diluted to a final 500 μl volume and separated on 12 ml of 10–50% sucrose gradient by ultracentrifugation at 230,500 × g for 2 h in an SW40 rotor (Beckman Coulter) at 4 °C. Fractions of 700 μl were collected using an ISCO gradient fractionation system and the OD254 was continuously recorded with a Foxy JR Fractionator (Teledyne ISCO) during the collection process.

### Size exclusion chromatography

HEK293 cells overexpressing 3xFlag-ZNF598 (20 × 10^6^) were collected by centrifugation and resuspended in 600 μl of lysis buffer containing 25 mM HEPES-KOH, pH 7.4, 150 mM KCl, 75 mM KOAc, 2 mM MgCl_2_, 0.5% NP40, supplemented with complete EDTA-free protease inhibitor cocktail and 1 mM NaF, 1 mM Na_3_VO_4_ and 1 mM β-glycerophosphate phosphatase inhibitor. The lysate was clarified by centrifugation at 15,000 × g for 10 min at 4 °C. 5 mg of the protein extract was brought to a total volume of 500 μl of lysis buffer (final concentration 10 μg μl^−1^) and directly loaded onto a 24 ml Superose 6 column (HR 10/300, GE Healthcare Life Sciences) pre-equilibrated with lysis buffer and run in the same buffer at a flow rate of 0.5 ml min^-1^. Molecular mass calibration was carried out by using the Gel Filtration HMW Calibration Kit (Healthcare Life Sciences, 28-4038-42).

### Fluorescence microscopy

Immunofluorescence and RNA-FISH experiments were performed following the protocol described previously^23^. For stress granule assays, HEK293 cells expressing 3xFlag-ZNF598 were grown on chamber slides. Arsenite was added to the cells at a final concentration of 400 μM and incubation was continued for 30 min at 37 °C. Chamber slides were hybridized overnight at 40 °C in hybridisation buffer containing 20 nM LNA-modified oligoT probe labelled with ATTO647N for detection of polyA and a cocktail of four different anti-28S rRNA LNA-modified oligodeoxynucleotides labelled with ATTO550 at 10 nM each for detection of 28S rRNA^23^. The slides were subsequently incubated for 1 h with anti-Flag antibody (Sigma, F3165) followed by incubation with a DAPI and Alexa Fluor 488-labelled goat anti-mouse IgG H+L (Thermo Fisher Scientific, A11001) for 1 h at RT. Images were recorded on the Olympus VS110 and processed using Visiopharm Integrated Systems Inc. software.

### PAR-CLIP

PAR-CLIP was performed using a single RNase A digestion step and using anti-Flag-M2 magnetic beads (Sigma, M8823) for IP as described previously^39^. PAR-CLIP cDNA libraries were sequenced on an Illumina HiSeq 2500 instrument, and data were analysed using the PAR-CLIP suite^39^. Reads mapping to mRNAs with d1 T-to-C sequence transitions and ≥20 nt were extracted and mapped to human genome using STAR-2.5.2a. For the metagene analysis, gencode gtf file (gencode.v19.chr_patch_hapl_scaff.annotation.gtf) was used to calculate the logarithm of average read coverage for 3′ UTR, CDS and 5′ UTR for the 2,000 mRNAs with highest coverage. Relative UTR and CDS sizes were calculated based in their average size in all mRNAs expressed in HEK293. Average coverage was calculated for each UTR and CDS independently according to the actual length of the region and number of bins (10 for 5′ UTR, 60 for CDS and 20 for 3′ UTR) and additionally represented in the upper panel as percent of total gene coverage. Reads mapping to tRNAs with d1 T-to-C sequence transitions and ≥20 nt were extracted and compared with tRNA sequencing (Gogakos, T & Tuschl, T, Characterizing expression and processing of precursor and mature human tRNAs by hydro-tRNAseq and PAR-CLIP, manuscript in preparation) data set obtained by hydro-tRNAseq^40^ for differential expression. The analysis was conducted with the R/Bioconductor package edgeR^41^ v. 3.14.0. The read counts were normalized using the weighted trimmed mean of M values^42^ and normalized for library size. The differences were tested using the Fisher's exact test and the read count variation was estimated using tagwise or common dispersion. Differences were considered significant below a false discovery rate of 5%. Reads mapping to rRNAs with d0, d1 T-to-C and d1 other and ≥15 nt were mapped independently using bowtie2 (ref. 43) to rRNA sequences, and bedtools^44^ were used to obtain the coverage across each rRNA.

### polyA mRNA-sequencing

Oligo(dT)-selected RNA was converted into cDNA for polyA mRNA-sequencing using the Illumina TruSeq RNA Sample Preparation Kit v2 according to the instructions of the manufacturer and sequenced on an Illumina HiSeq 2500 platform using 100 nt single-end sequencing.

### Co-IP and mass spectrometry (IP/MS)

Cell lines expressing 3xFlag and 3xFlag-ZNF598 from one 15-cm plate (∼20 × 10^6^) were lysed in NP40 lysis buffer composed of 50 mM HEPES-KOH, pH 7.5, 150 mM KCl, 2 mM MgCl_2_, 2% v/v NP40, 0.5 mM DTT, 1 × complete EDTA-free protease inhibitor cocktail and 1 × PhosStop (Roche, 04906837001) and the Flag-tagged protein was immunoprecipitated from the lysate with anti-FLAG-M2 magnetic beads. Magnetic beads were washed three times with washing buffer composed of 50 mM HEPES-KOH, pH 7.5, 300 mM KCl, 2 mM MgCl_2_, 0.5% v/v NP40 and three times with high-salt washing buffer composed of 50 mM HEPES-KOH, pH 7.5, 500 mM KCl, 2 mM MgCl_2_, 0.5% v/v NP40. The immunoprecipitates were eluted with Flag peptide and separated on NuPAGE Novex 4–12% Bis-Tris protein gels (Thermo Fisher Scientific, NP0322BOX), which were run shortly with the dye front 12 mm from the loading pocket. The experiment was performed for three biological replicates per condition. The protein bands were excised, carefully washed and followed by reduction (DTT final concentration of 5 mM for 30 min at 55 °C) and alkylation (iodoacetamide final concentration of 2 μg ml^−1^ for 15 min at room temperature). Proteins were digested overnight with Endopeptidase Lys-C (Wako) and trypsin (Sequencing Grade, Promega). Peptides were extracted, desalted^45^ and analysed by nano LC-MS/MS (Dionex Ultimate 3000 coupled to a Q-Exactive Plus, Thermo Fisher Scientific). Data were processed using MaxQuant v. 1.5.3.28 (ref. 46). Proteins quantitated in two out of three biological replicates for at least one condition and with Welch’s *t*-test false discovery rate <5% were analysed using the statistical software Perseus^47^.

### Co-IP assays

Parental Flp-In T-REx 293 cells (6 × 10^6^ cells grown in a 10-cm plate) were transiently transfected with pcDNA5.A-3xFlag-ZNF598 and control EV (3xFlag) using Lipofectamine 2000 according to the manufacturer’s instructions. After 24 h, cells were washed twice with cold PBS and collected in 1 ml of cold lysis buffer composed of 40 mM HEPES-KOH, pH 7.5, 0.3% CHAPS, 120 mM NaCl, 1 mM EDTA supplemented with complete EDTA-free protease inhibitor cocktail, and RNase A (10 μg/ml), and incubated for 30 min on ice. Lysates were cleared by centrifugation at 20,000 × g for 15 min at 4 °C. The protein concentration of lysates was quantified using the Bradford assay and 1 mg of lysate protein was used for IP. Before IP, the lysates were cleared again by incubating with 50 μl of 50% protein G agarose fast flow beads (EMD Millipore, 16–266) for 2 h at 4 °C with gentle agitation. The pre-cleared lysates were centrifuged at 3,000 × g for 1 min at 4 °C and the supernatant was incubated with 1 μl of anti-Flag M2 antibody in 1 ml total volume on an end-over-end rotator for 3 h at 4 °C. Subsequently, 50 μl of 50% protein G agarose beads were added to the lysate/antibody and the mixture was incubated at 4 °C overnight on an end-over-end rotator. Beads were washed three times with 500 μl of lysis buffer and the immunoprecipitated complex was eluted from the beads by boiling in 30 μl of 2 × SDS loading buffer composed of 100 mM Tris-HCl, pH 6.8, 4% w/v SDS, 0.2% bromophenol blue, 20% v/v glycerol and 200 mM DTT for 10 min at 65 °C.

### Ubiquitin remnant immunoaffinity profiling

Proteome-wide ubiquitination sites were identified by using the PTMScan Ubiquitin Remnant Motif (K-ε-GG) Kit (Cell Signaling Technologies, 5562). Ubiquitinated peptides were enriched and identified by immunoprecipitation using a bead-conjugated monoclonal antibody generated against the sequence CXXXXXXK-ε-GGXXXXXX, where X is any amino-acid except cysteine and tryptophan, and K-ε-GG is a di-glycine moiety bound to the ε amino group of a lysine residue. Enrichment of ubiquitinated peptides in conjunction with liquid chromatography (LC) tandem mass spectrometry (MS/MS; EasyLC 1200 coupled to a Fusion Lumos operated in HCD high/high mode, Thermo Fisher Scientific) was performed according to the manufacturer’s instructions. In brief, for each experiment ∼1–2 × 10^8^ cells were grown to 90% confluency. Cells were harvested, washed with 1 × PBS, and lysed in 10 ml of freshly prepared urea lysis buffer (20 mM HEPES-KOH, pH 8.0, 9 M urea, 1 mM Na_3_VO_4_, 2.5 mM sodium pyrophosphate, 1 mM β-glycerophosphate, 1 mM iodoacetamide). Using a microtip, the cell lysate was sonicated by three 15 s bursts at 15 W. The lysate was cleared by centrifugation for 15 min at 20,000 × g at room temperature. The cleared supernatant contained about 20 mg of total protein and was subjected to reduction (DTT final concentration of 5 mM for 30 min at 55 °C) and alkylation (iodoacetamide final concentration of 2 μg ml^−1^ for 15 min at room temperature) followed by overnight trypsinization (TPCK Treated, Worthington Biochemical Corporation). Peptides were desalted and purified using a Sep-Pak C18 column (WAT051910, Waters Corporation). Peptides were eluted with 20 ml Solvent B (0.1% TFA, 40% acetonitrile), frozen in liquid nitrogen, and lyophilized for 2 days to assure complete TFA removal. For immunoaffinity purification, the lyophilized peptides were dissolved in 1.4 ml IAP buffer containing 50 mM MOPS-NaOH, pH 7.2, 10 mM Na_2_HPO_4_, 50 mM NaCl. The peptide solution was added to a microtube of the anti-K-ε-GG motif antibody beads and immunoprecipitated on a rotator for 2 h at 4 °C. After IP, the bead slurry was centrifuged at 2,000 × g for 30 s and the supernatant was removed. The beads were washed three times with 1 ml of IAP buffer followed by two washes with 1 ml 1 × PBS. Peptides were eluted in 100 μl of 0.15% TFA, concentrated and desalted by stage tip chromatography, and subjected to nano LC-MS/MS analysis in technical replicate. LC-MS/MS data were queried against a Uniprots Human database (March 2016) using MaxQuant v. 1.5.3.28.

### Sequence alignment and domain analysis

Amino-acid sequences were obtained from the NCBI database. ZNF598 proteins: human, NP_835461.2; mouse, NP_898972.1; *Xenopus*, NP_001119543.1; *Drosophila*, NP_611932.2; *Arabidopsis*, NP_566094.2: *S. cerevisiae*, NP_010552.3. UBE2D family proteins: human UBE2D1 protein, NP_003329.1; human UBE2D2, NP_003330.1; human UBE2D3, NP_871621.1; human UBE2D4, NP_057067.1. Trim23 proteins: human, NP_001647.1; mouse, NP_109656.1; *Xenopus*, XP_002934252.2. CBLL1 proteins: human, NP_079090.2; human ZNF645, NP_689790.1; mouse, NP_001240776.1; *Xenopus*, NP_001123714.1; *Drosophila*, NP_001260593.1. Protein sequence alignments were carried out using PRALINE^48^,^49^ (http://www.ibi.vu.nl/programs/pralinewww/). Conservation analyses were carried out using Clustal Omega (http://www.clustal.org/omega/) in combination with Trex Newick Viewer (http://www.trex.uqam.ca/index.php?action=newick&project=trex) to obtain the radial tree. Domains analyses were performed via Interpro (≤http://www.ebi.ac.uk/interpro/). Disorder profile of proteins was mapped via DISOPRED3 (ref. 50).

### Data availability

The NCBI SRA accession numbers for the sequencing data reported in this paper are SRR5137268 and SRR5137269 for ZNF598 PAR-CLIP experiments and SRR5137268 for HEK293 polyA mRNA-Seq. Supplementary Data are also available on https://rnaworld.rockefeller.edu/ZNF598. The additional data that support the findings of this study are available from the corresponding author upon request.

## Additional information

**How to cite this article:** Garzia, A. *et al*. The E3 ubiquitin ligase and RNA-binding protein ZNF598 orchestrates ribosome quality control of premature polyadenylated mRNAs. *Nat. Commun.* **8**, 16056 doi: 10.1038/ncomms16056 (2017).

**Publisher’s note:** Springer Nature remains neutral with regard to jurisdictional claims in published maps and institutional affiliations.

## Supplementary Material

Supplementary Information

Supplementary Data 1

Supplementary Data 2

Supplementary Data 3

Supplementary Data 4

Supplementary Data 5

Supplementary Data 6
