# Supplementary material for: The E3 ubiquitin ligase and RNA-binding protein ZNF598 orchestrates ribosome quality control of premature polyadenylated mRNAs
Source: Nat Commun. 2017 Jul 7;8:16056. doi: 10.1038/ncomms16056 (PMC5504347; doi:10.1038/ncomms16056)
Supplement: Supplementary Information [file ncomms16056-s1.pdf]

Title of file for HTML: Supplementary Information
Description: Supplementary Figures

Title of file for HTML: Supplementary Dataset 1
Description: Mapping statistic and sequence read report resolved by RNA category of ZNF598 PAR-CLIP datasets. The number of extracted reads mapping perfectly (d0), with one mismatch (d1), or 2 mismatches (d2) to the human transcriptome were resolved by read length. Related to Figure 2a.

Title of file for HTML: Supplementary Dataset 2
Description: tRNA abundance and PAR-CLIP reads. Related to Figure 2e.

Title of file for HTML: Supplementary Dataset 3
Description: Transcript abundance of ZNF598, ribosomal proteins, and genes involved in the RQC pathway.

Title of file for HTML: Supplementary Dataset 4
Description: Occurrence of repeated poly-purine codons within unperturbed coding sequence (CDS) in HEK293 cells. Related to Supplementary Figure 11.

Title of file for HTML: Supplementary Dataset 5
Description: List of differentially ubiquitinated proteins in ZNF598-OE and ZNF598-KO cells. Related to Figure 4a.

Title of file for HTML: Supplementary Dataset 6
Description: List of ZNF598-interacting proteins in HEK293 cells detected by immunoprecipitation followed by mass spectrometry (IP/MS). Related to Figure 4b and Supplementary Figure 15.

Supplementary Figure 1

a

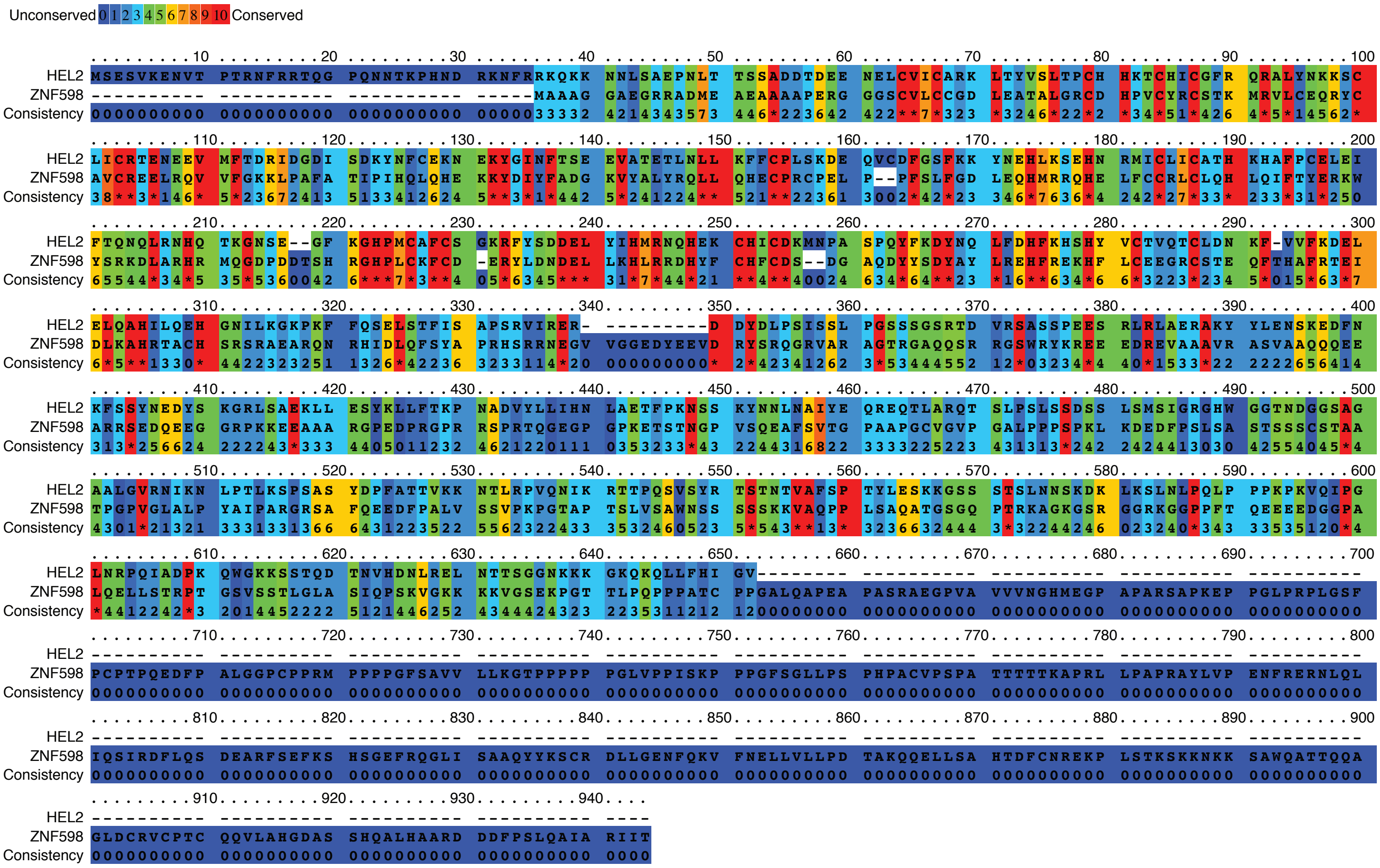

b

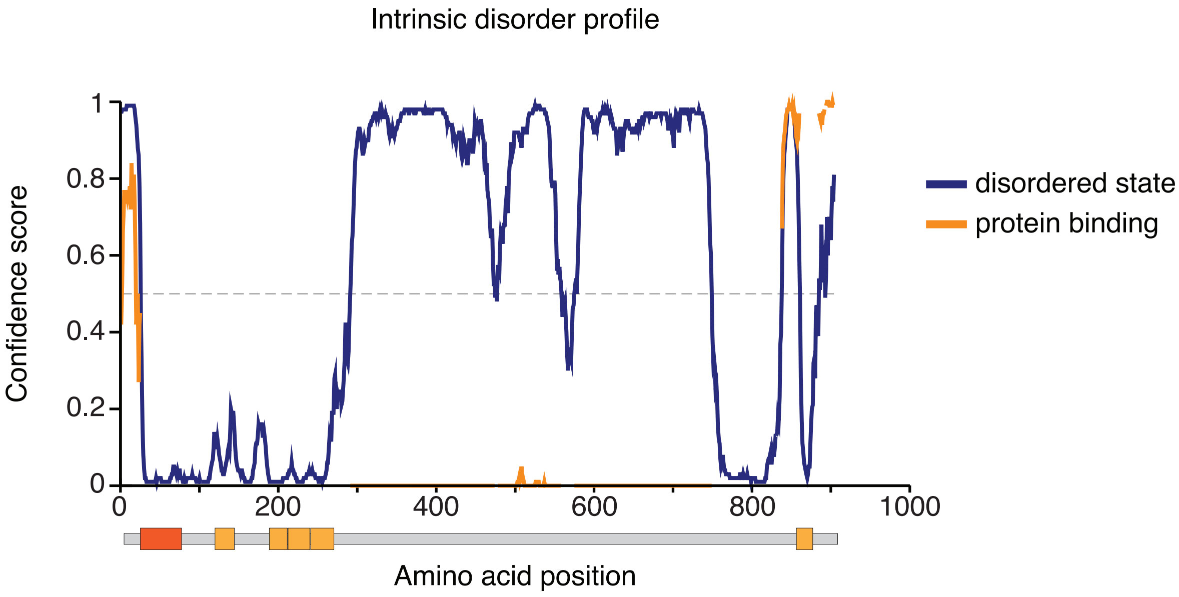

Supplementary Figure 1. Comparison of ZNF598 and Hel2 protein sequences and domain structures. (**a**) PRALINE alignment of human ZNF598 and yeast Hel2 protein sequences. Numbers and background colors indicate the conservation scores. (**b**) DISOPRED3 disorder profile of human ZNF598, showing the disorder confidence levels against the sequence positions as a solid blue line. The grey dashed horizontal line marks the threshold above which amino acids are regarded as disordered. For disordered residues, the orange line shows the confidence of disordered residues involved in protein-protein interactions.

Supplementary Figure 2

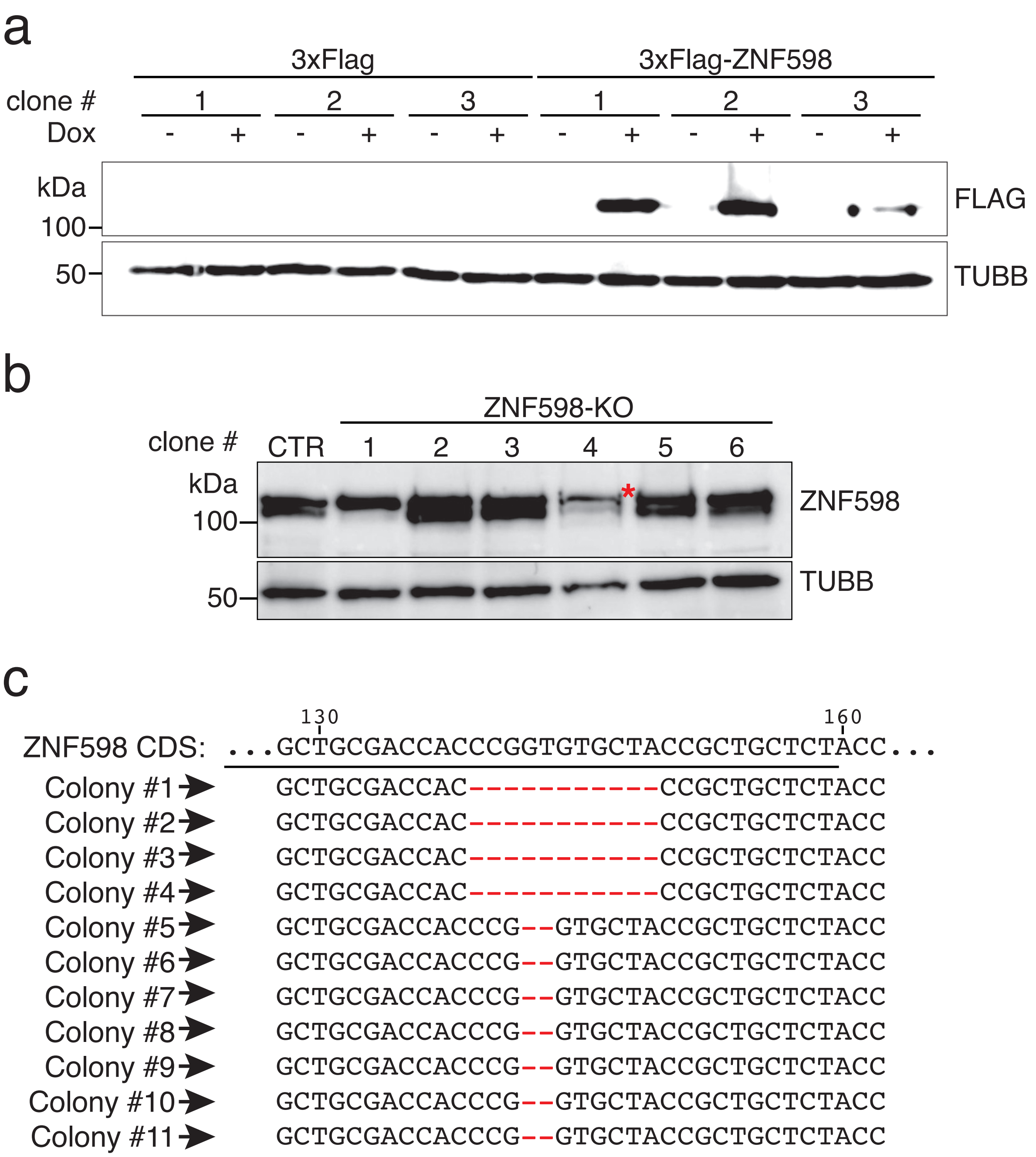

Supplementary Figure 2. Overexpression and CRISPR-CAS9-mediated depletion of ZNF598 in HEK293 cells.

(**a**) Screen for stable Flp-In T-REx 293 cell clones inducible expressing 3xFlag-tagged ZNF598. Clone 1 was selected for further experiments. (**b**) Western blot with ZNF598 antibody (Abcam) of 6 different CRISPR-CAS9-induced ZNF598-knockout colonies. Clone 4 was used in further experiments. CTR represents the parental Flp-In T-REx 293 cells. * Indicates a non-specific band (Abcam antibody). (**c**) Sequence alignment of 11 cloned PCR products amplified from the genomic segment of ZNF598 targeted by sgRNA of clone 4 revealing disruption of the coding sequence.

Supplementary Figure 3

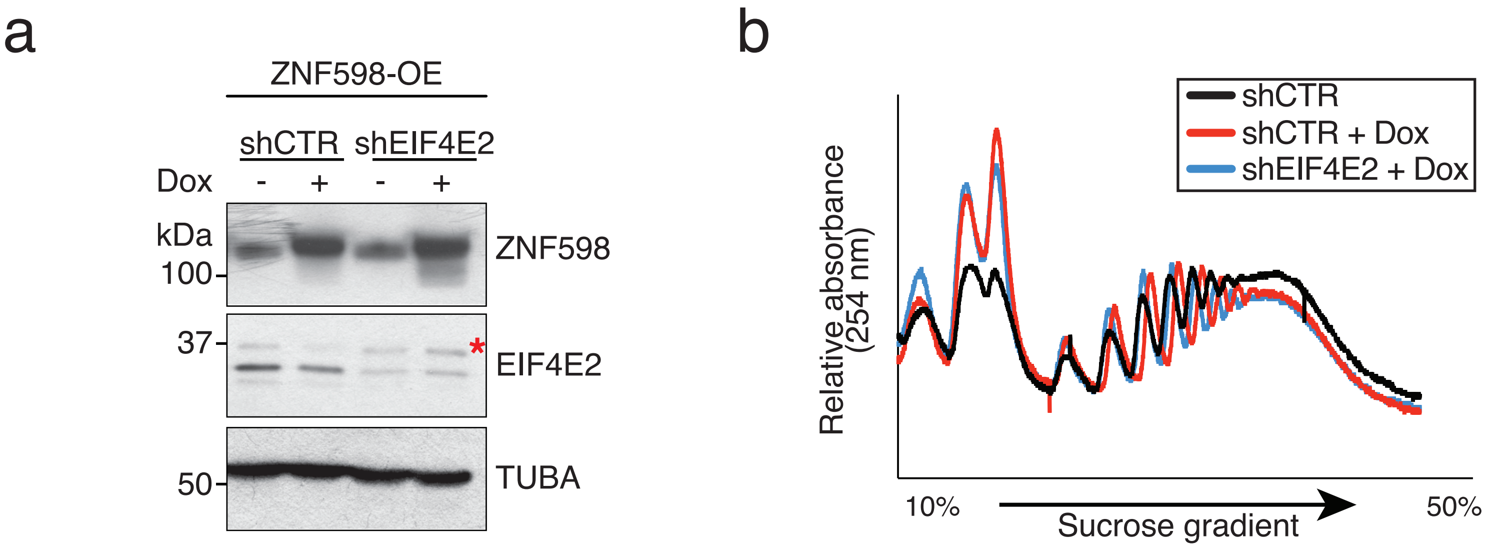

Supplementary Figure 3. Depletion of EIF4E2 did not abrogate the translational repression induced by ZNF598 overexpression.

(**a**) Western blot analysis of ZNF598-OE cells stably expressing the non-targeting control shRNA (shCTR) or shRNA targeting EIF4E2 (shEIF4E2). * indicates a non-specific band. (**b**) Polysome profiles of the cells described in (a).

Supplementary Figure 4

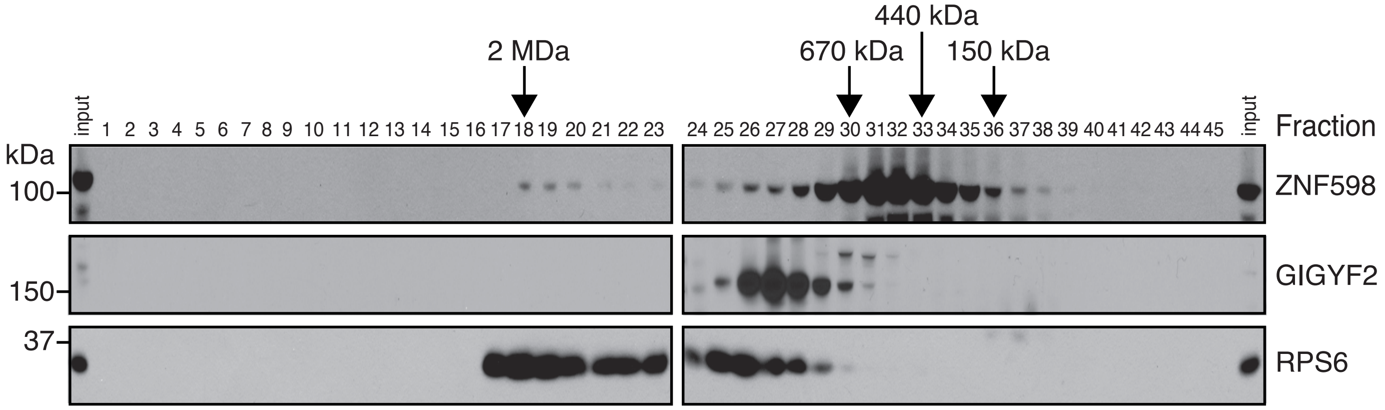

Supplementary Figure 4. Co-fractionation of ZNF598 with ribosomal proteins in a >2 MDa complex. ZNF598-OE cell lysate was subjected to size-exclusion and fractions were analyzed by Western blotting for the presence of the indicated proteins. The elution position of the molecular mass markers is shown.

Supplementary Figure 5

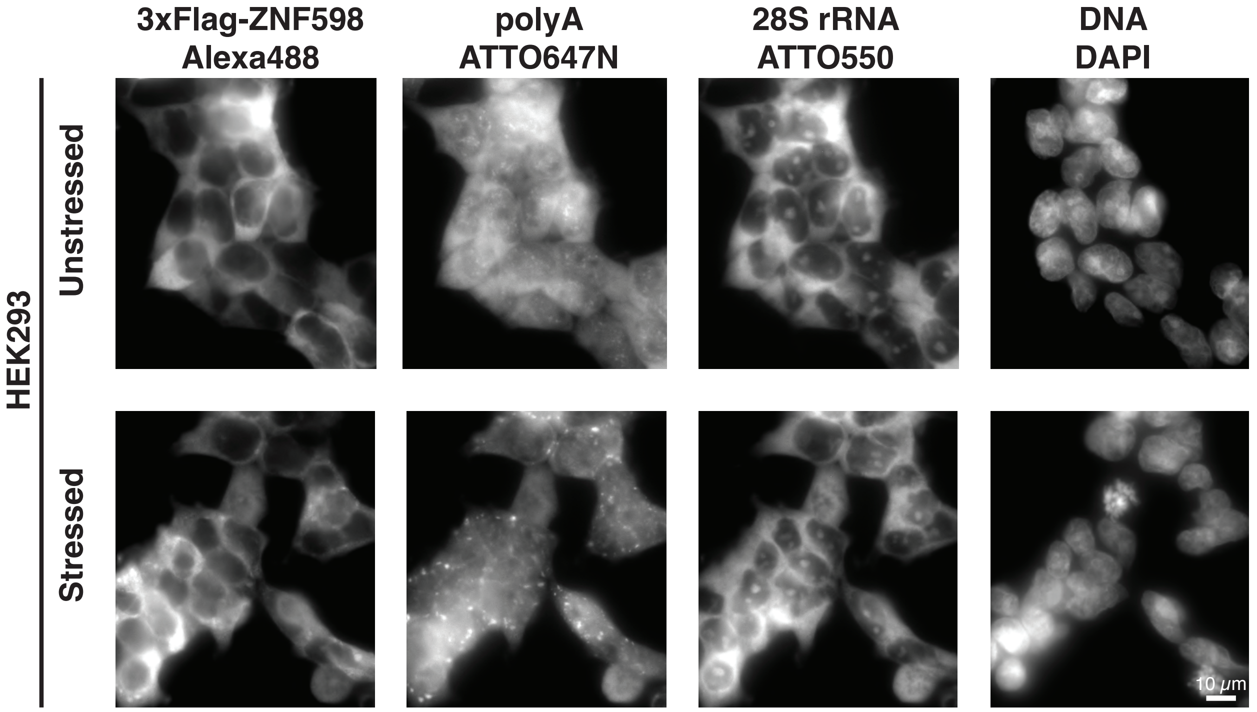

Supplementary Figure 5. ZNF598 is a cytosolic protein, which is absent from arsenite-induced stress granule.
Simultaneous detection of ZNF598, polyA mRNAs and 28S rRNA in ZNF598-OE cells by combined multicolor RNA-FISH and immunofluorescence (IF). Cells were exposed to 400 μM sodium arsenite for 30 min or left untreated. PolyA mRNAs and 28S rRNA were detected by hybridization to ATTO647N and ATTO550 fluorescently labeled probes, respectively. ZNF598 was detected by IF using primary anti-Flag, and corresponding fluorescently labeled (Alexa488) secondary antibody. Nuclei were visualized by DAPI staining.

Supplementary Figure 6

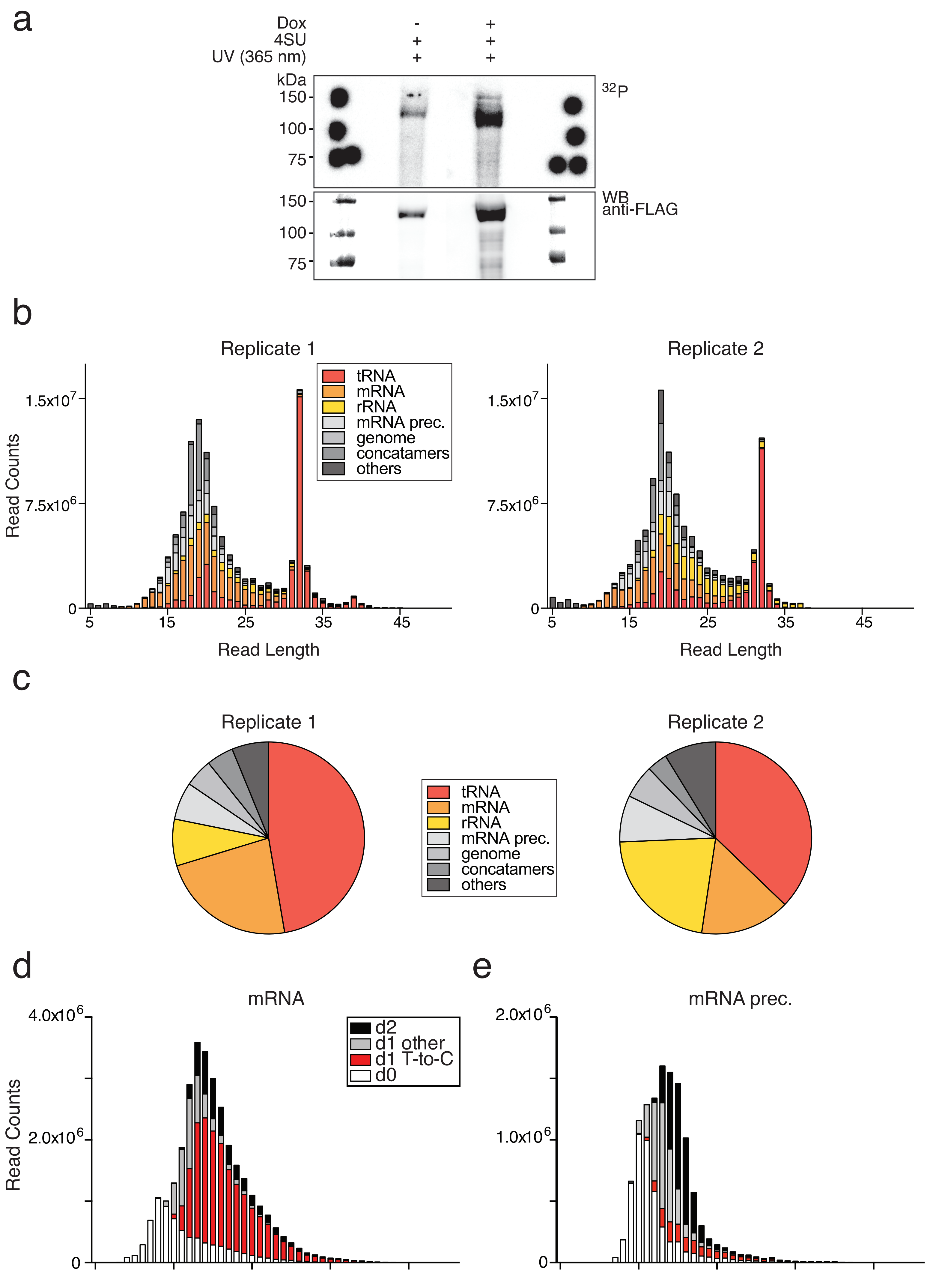

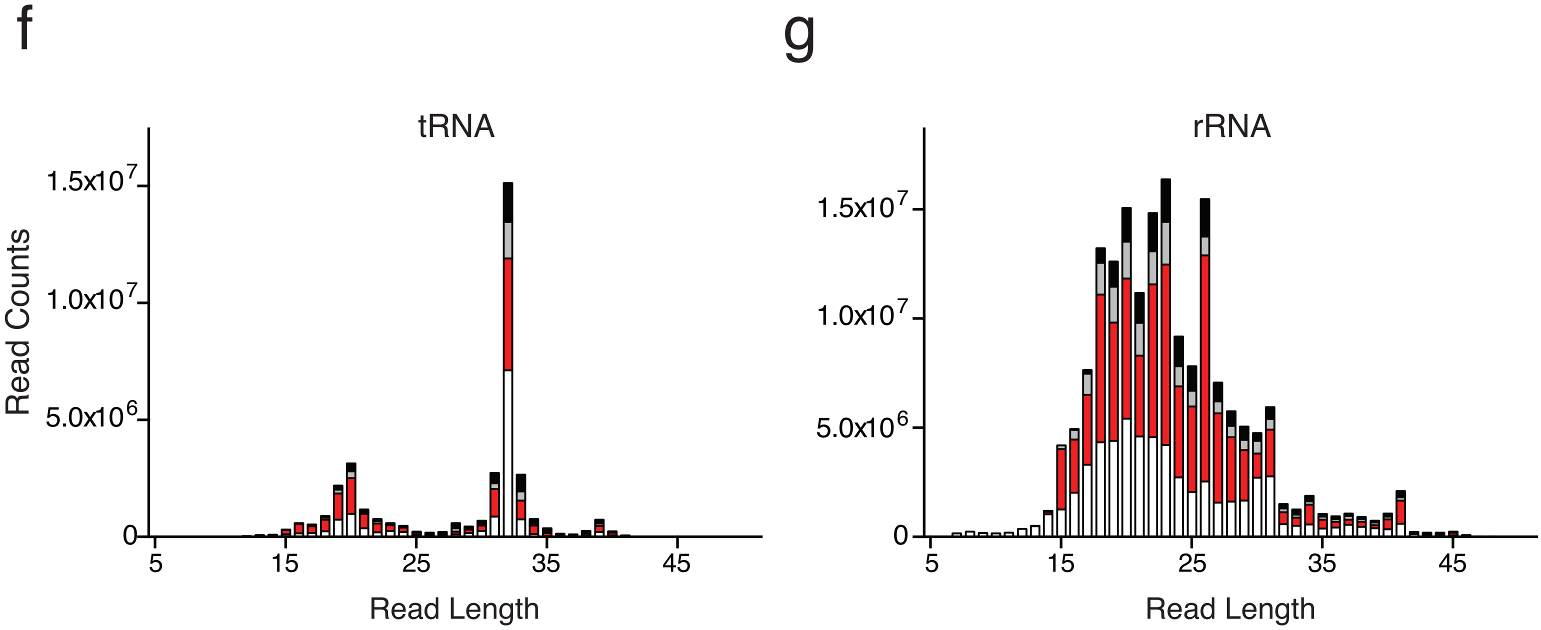

Supplementary Figure 6. Composition of replicate ZNF598 PAR-CLIP cDNA libraries using the PAR-CLIP suite pipeline.

(**a**) Upper panel: Autoradiograph of crosslinked and 5' radiolabeled RNA-3xFlag-ZNF598 immunoprecipitate separated by SDS-PAGE following 4SU PAR-CLIP. Lower panel: Western blot analyses of the crosslinked immunoprecipitate using the anti-Flag antibody. (**b**) Composition of ZNF598 PAR-CLIP sequence reads from 2 replicates. Reads mapping to each RNA category with up to 2 mismatches are represented by stacked bar graphs and resolved by length of adapter-extracted sequence. (**c**) Pie-chart representation of the composition of ZNF598 PAR-CLIP sequence reads from the 2 replicates by RNA categories mapping to each RNA category with up to 2 mismatches. (**d-g**) Composition of the most abundant RNA categories assigned as error distance 0 (d0, white), error distance 1 (d1 T-to-C (red) and d1 other than T-to-C (light grey), or error distance 2 (d2), including insertions, or deletions.

Supplementary Figure 7

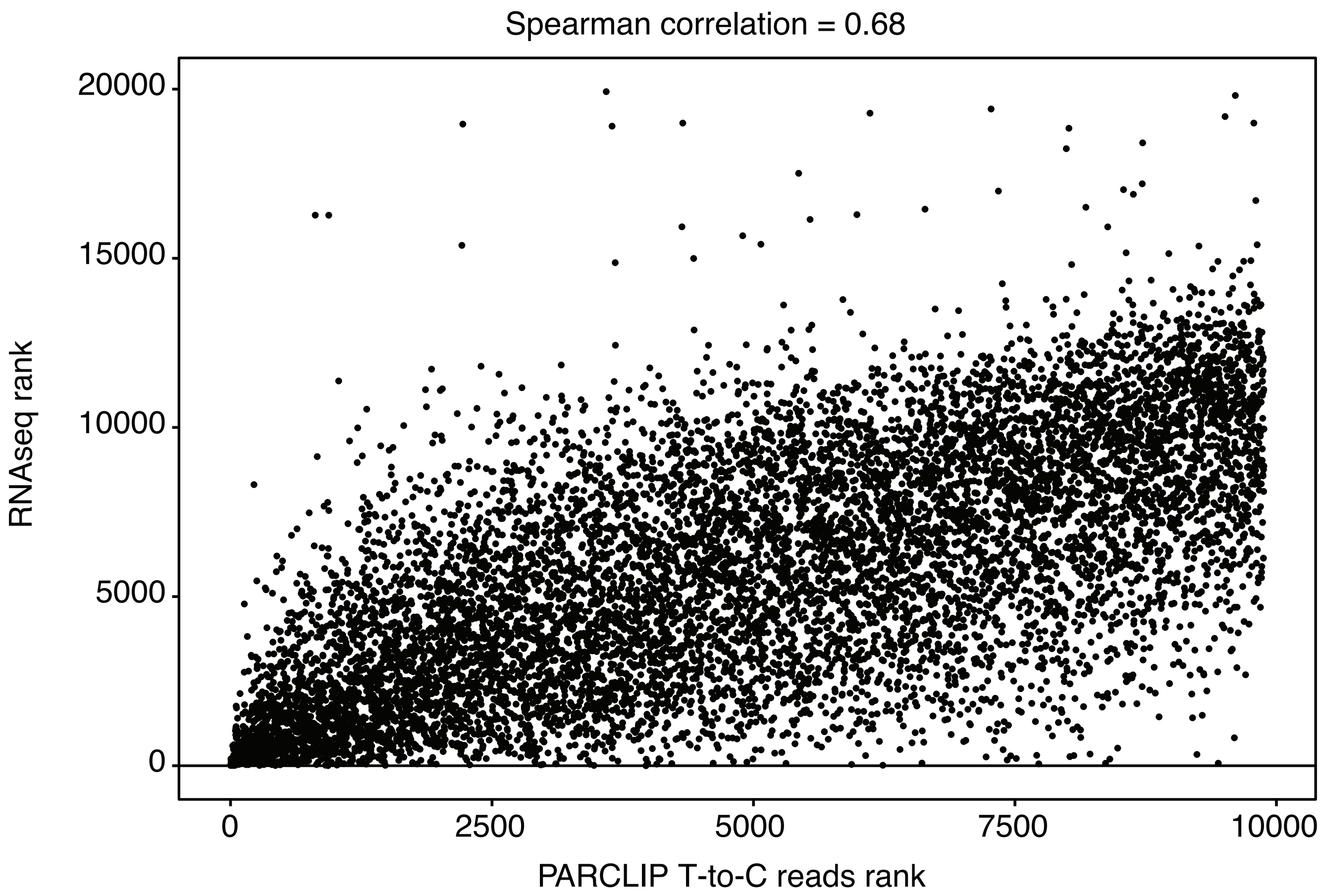

Supplementary Figure 7. Correlation between mRNA abundance and ZNF598 PAR-CLIP reads. Correlation between ranked abundance of each mRNA from a polyA mRNA-Seq in HEK293 and ranked abundance of the PAR-CLIP reads (d1 T-to-C) for the same mRNAs. The Spearman rank-order correlation coefficient is shown.

Supplementary Figure 8

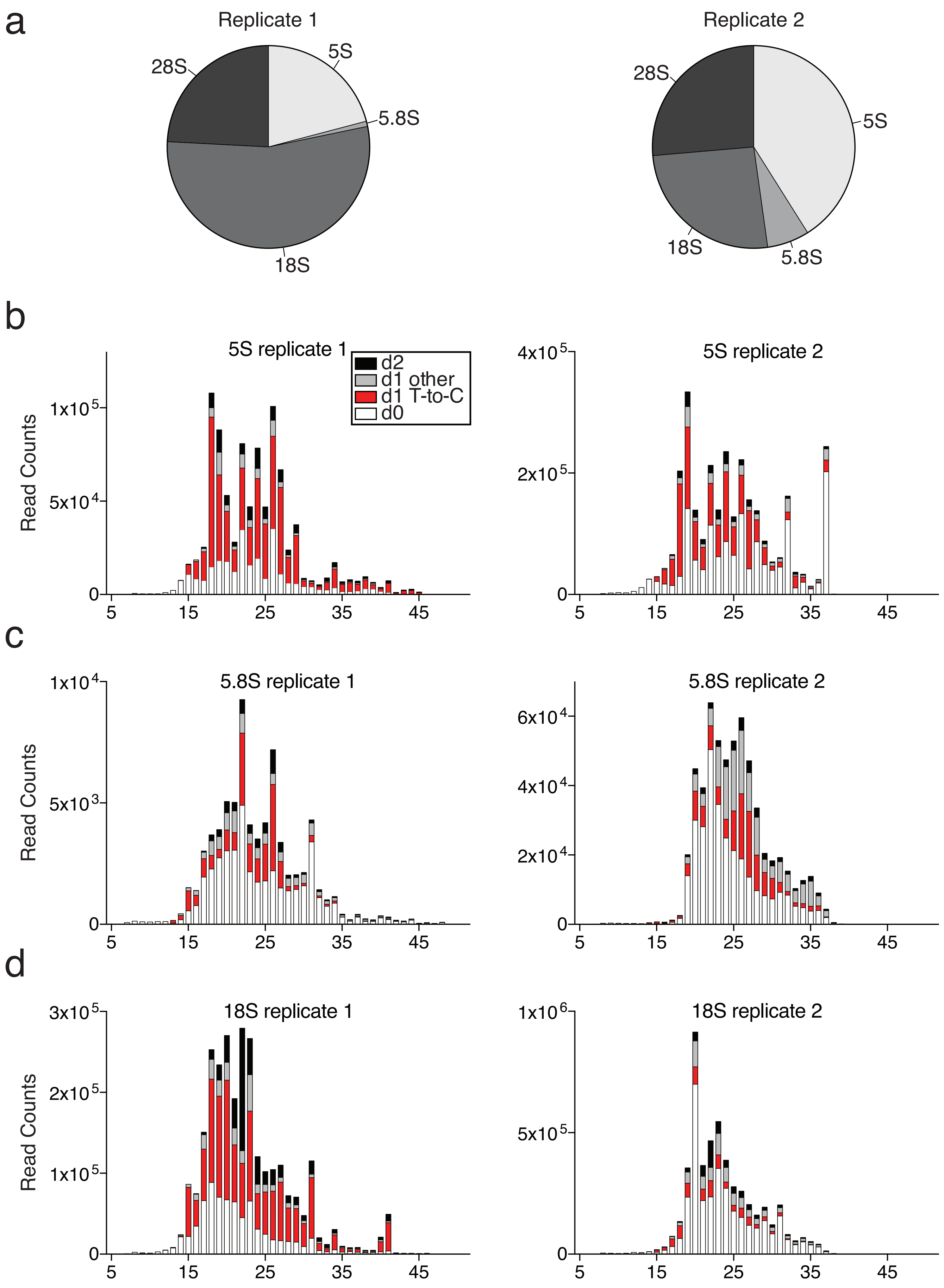

e

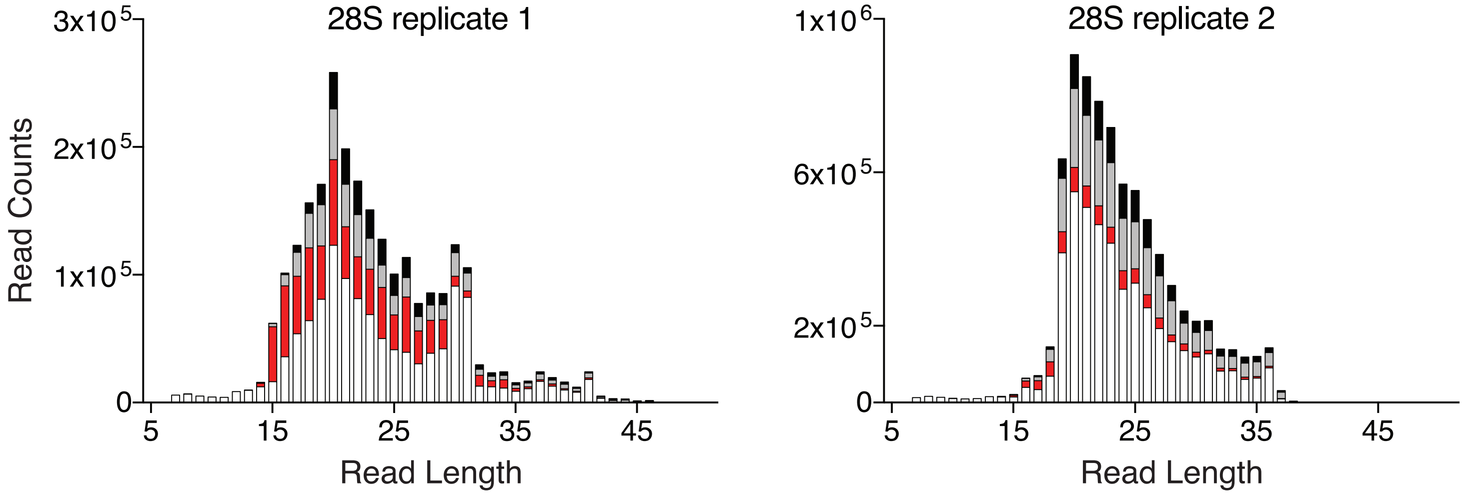

Supplementary Figure 8. Overview of ZNF598 PAR-CLIP sequence reads derived from major, nuclear encoded rRNAs.
(**a**) Composition of ZNF598 PAR-CLIP sequence T-to-C reads annotated as rRNAs from each replicate. (**b-e**) Composition of each of the different rRNAs assigned as error distance 0 (d0, white), error distance 1 T-to-C (d1 T-to-C, red) and error distance 1 other than T-to-C (d1 other, light grey), or error distance 2 (d2, dark grey), including insertions, or deletions.

Supplementary Figure 9

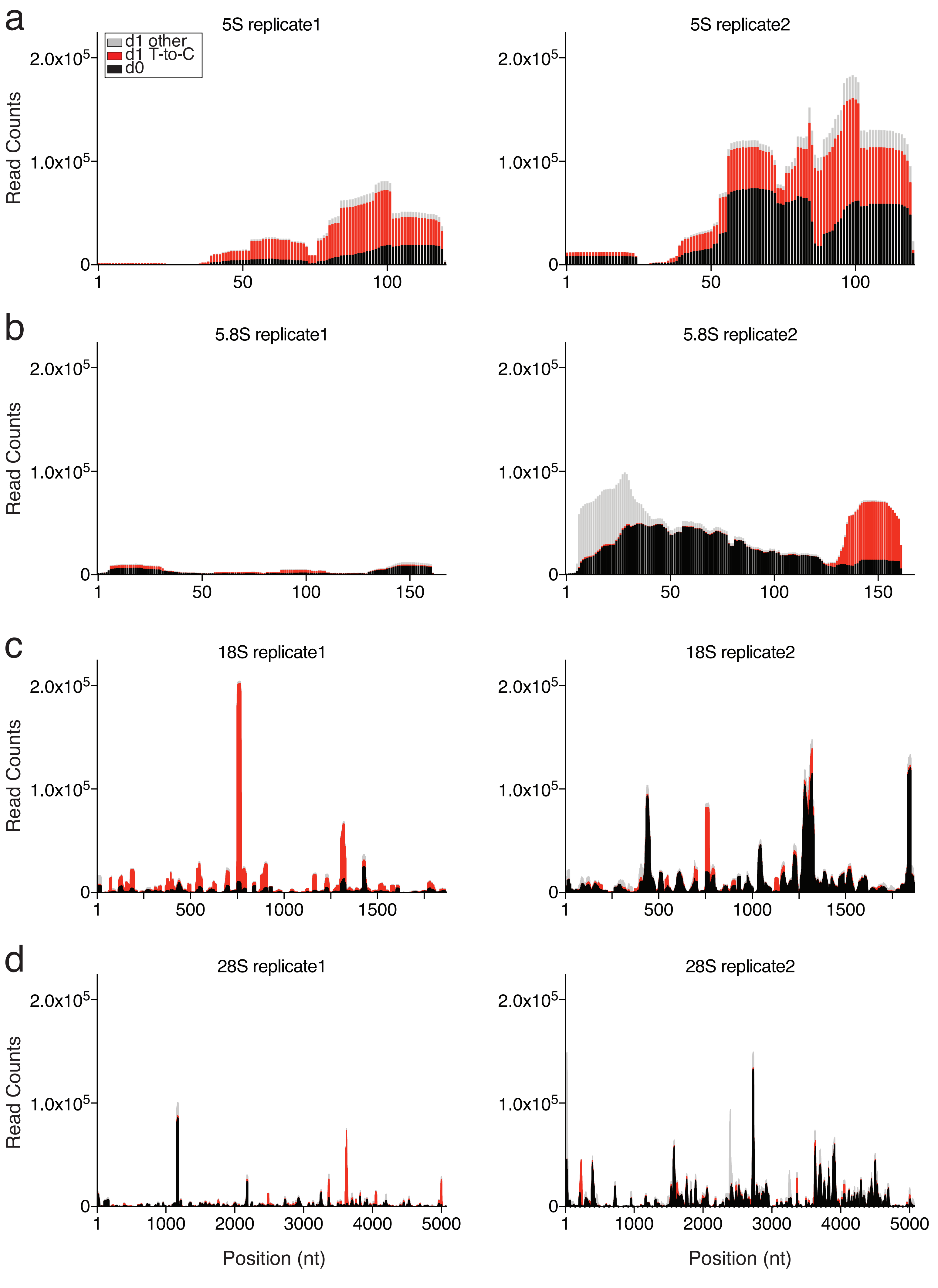

Supplementary Figure 9. Sequence read alignment of ZNF598 PAR-CLIP reads to each member of the major, nuclear encoded rRNAs.

ZNF598 PAR-CLIP sequence reads of 2 replicates across 5S (**a**), 5.8S (**b**), 18S (**c**) and 28S (**d**). rRNA assigned as d0 (white), d1 T-to-C (red) and d1 other than T-to-C (light grey), or d2.

Supplementary Figure 10

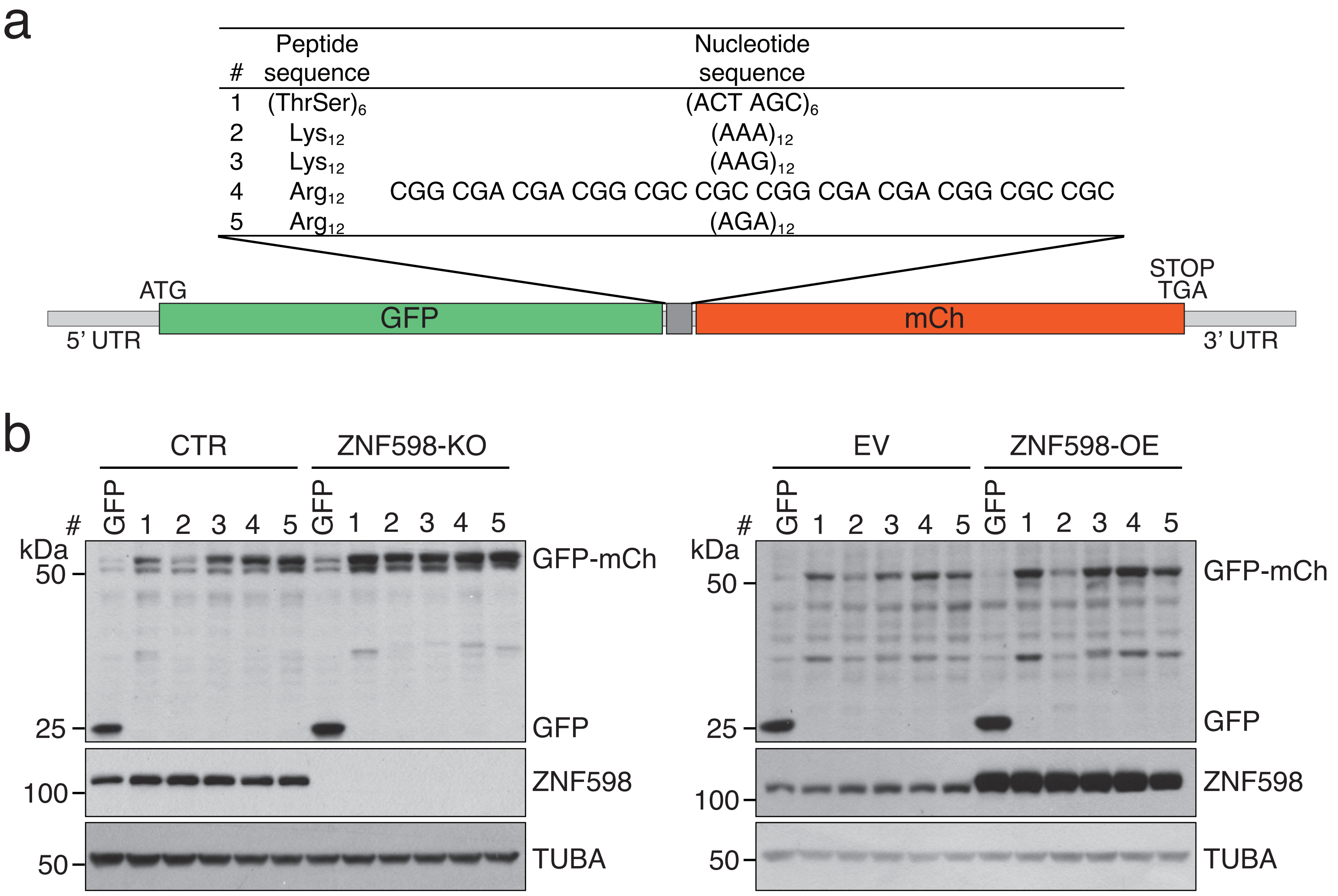

Supplementary Figure 10. Reporter assay for analysis of ribosome stalling at premature polyA sequences.

(**a**) Schematic diagram of the fluorescent protein reporter constructs containing a polybasic tract sandwiched between the fluorescent proteins GFP and mCherry (mCh). (ACT AGC)6 [(ThrSer)6] was used as control encoding a neutrally charged amino acid tract. (**b**) Western blot analysis using GFP antibody to detect the expression of transiently transfected reporters indicated in (a) in CTR or ZNF598-KO (left) and ZNF598-OE cells or EV (right). A plasmid expressing only GFP was used as control.

Supplementary Figure 11

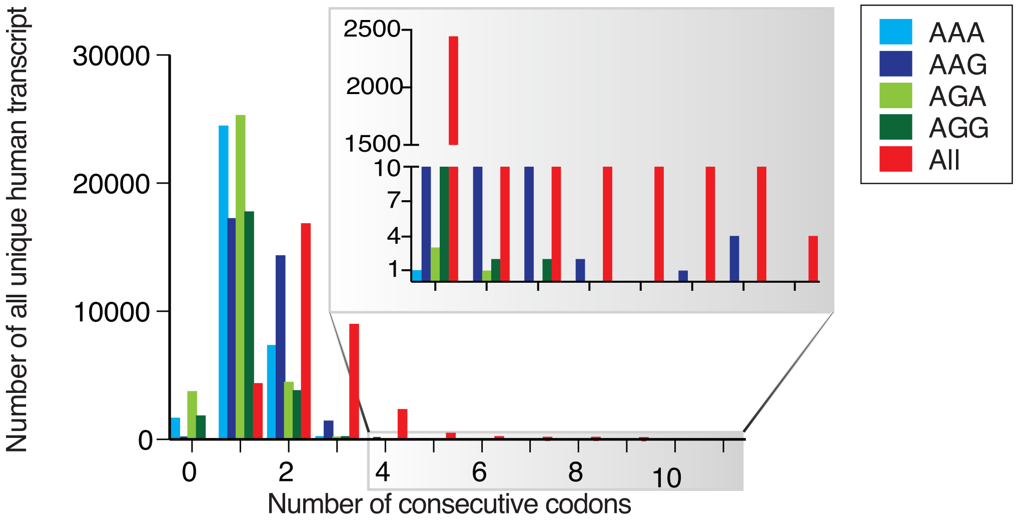

Supplementary Figure 11. Consecutive AAA codons are rare in the human transcriptome. Frequency of unique mRNA transcripts in the human transcriptome and increasing number of consecutive in-frame poly-purine codon stretches encoding lysine or arginine amino acids, respectively. “All” represents the sum of all indicated poly-purine codon stretches.

Supplementary Figure 12

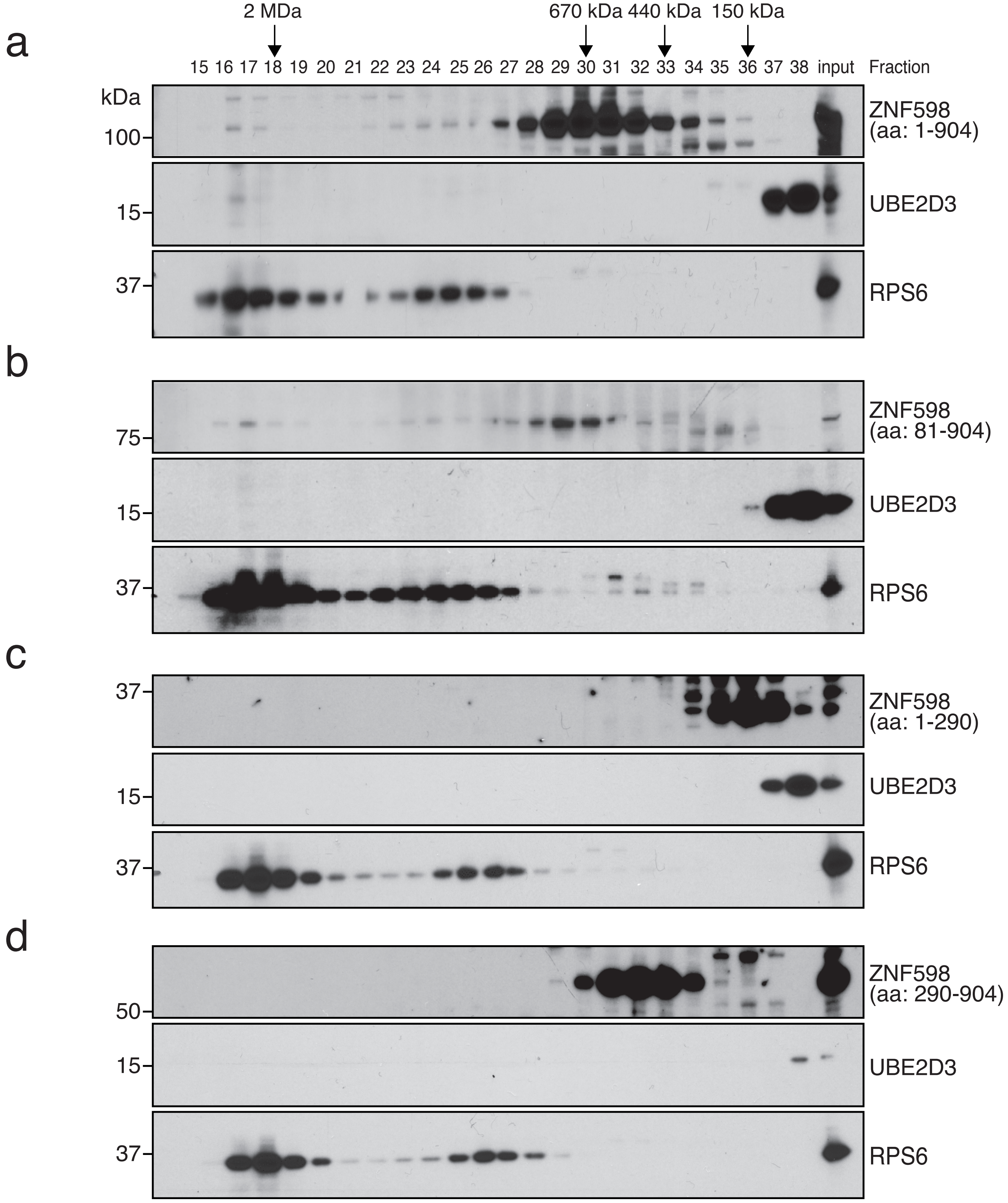

Supplementary Figure 12. The E3 ligase RING domain of ZNF598 is not required for binding to ribosomes.

Size-exclusion chromatography and Western blot analysis for the presence of the full length or truncated ZNF598 was performed as indicated in Supplementary Figure 4. Flag/HA-ZNF598 was detected using primary anti-Flag antibody. Co-fractionation of ZNF598 with a >2 MDa complex was observed irrespective of the presence of the RING domain.

Supplementary Figure 13

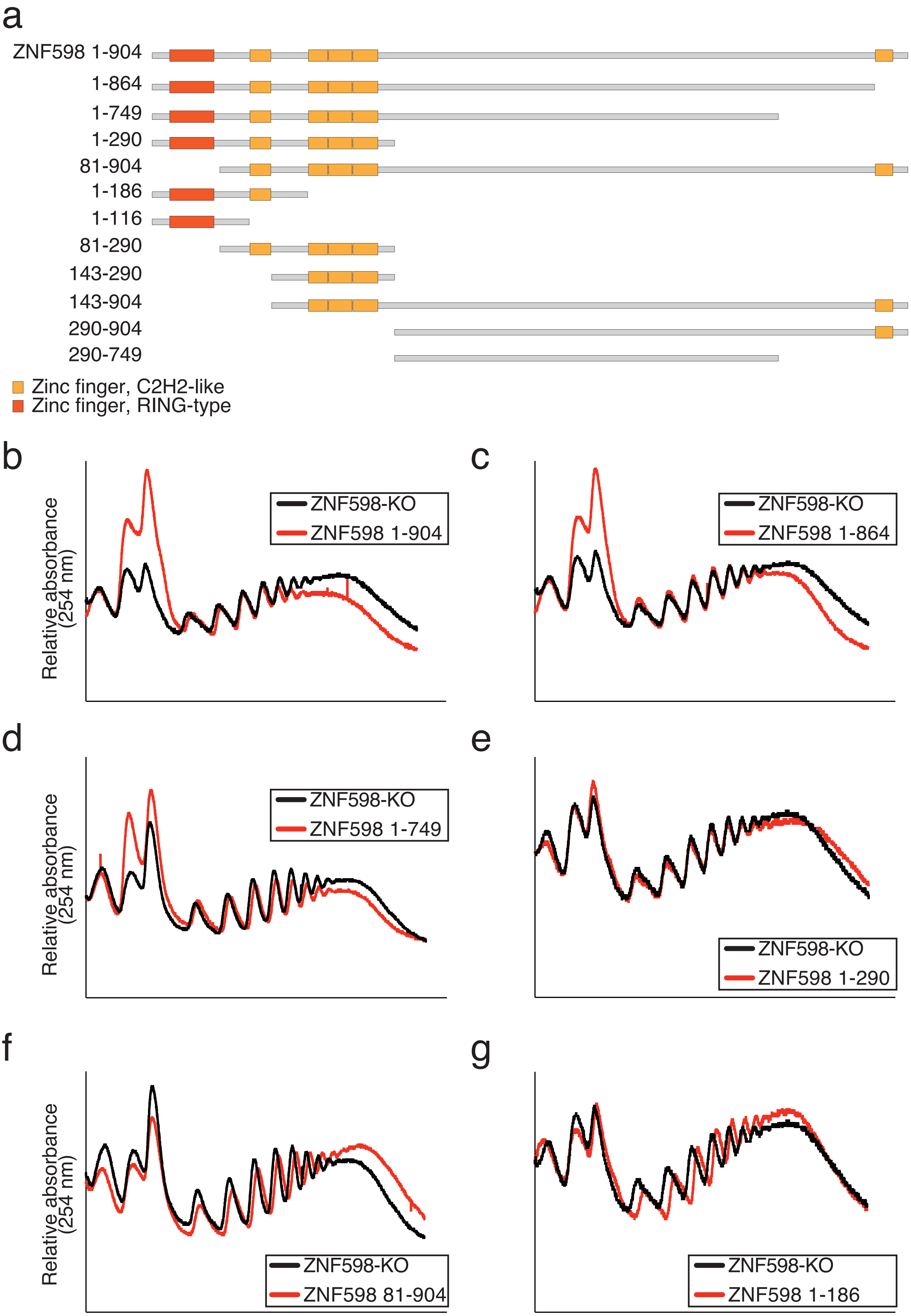

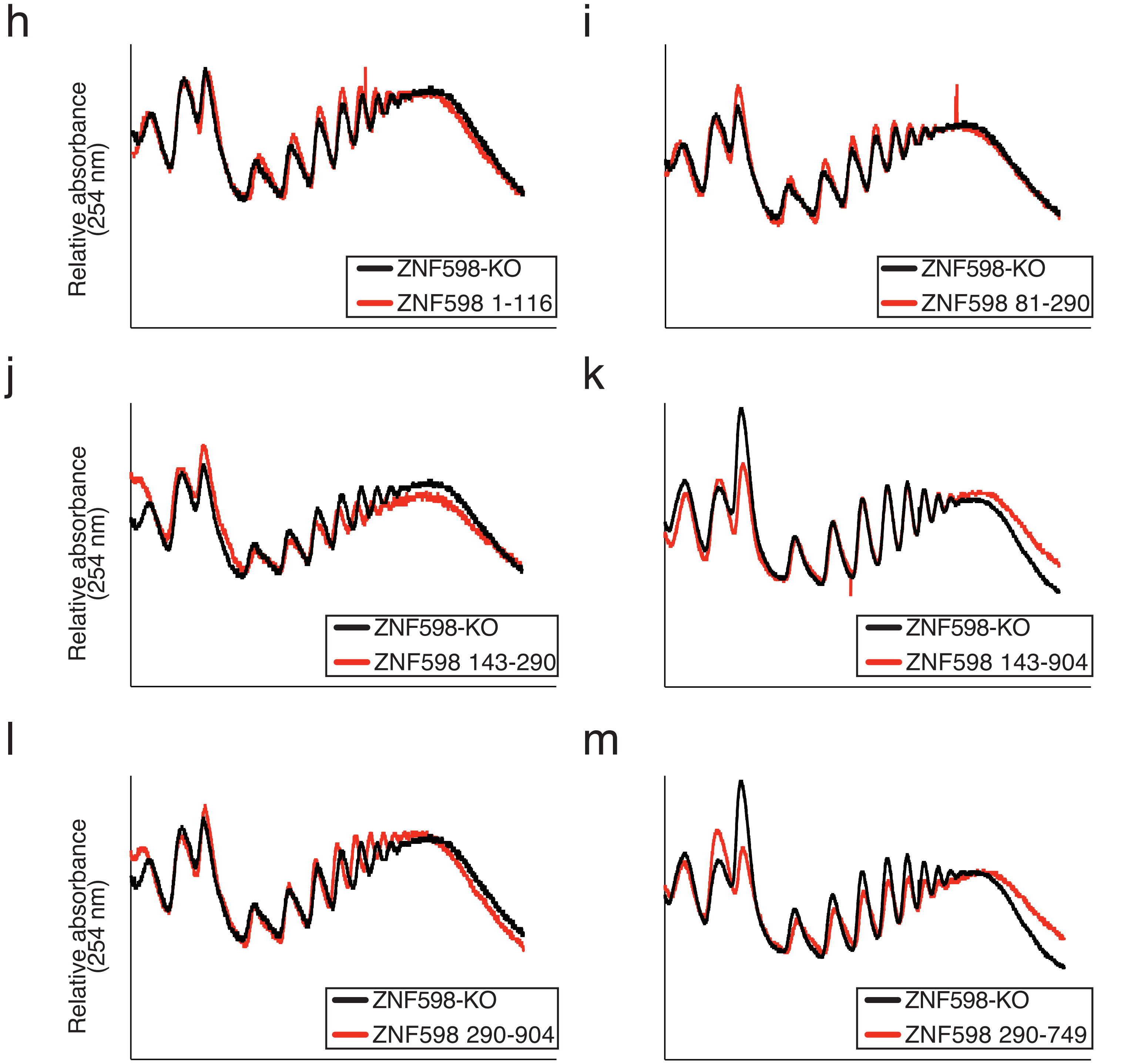

Supplementary Figure 13. The N-terminal RING domain, N-terminal C2H2-type zinc finger motifs and the unstructured domain of ZNF598 are essential for translation repression.
(**a**) Domain structures of full-length and truncated ZNF598 with numbers indicating amino acid positions. (**b-m**) Polysome profiles of inducible full-length or truncated ZNF598 clonal lines described in (a). Non-selective media was used for all cell lines to avoid using different media for ZNF598-KO and complemented cell lines.

Supplementary Figure 14

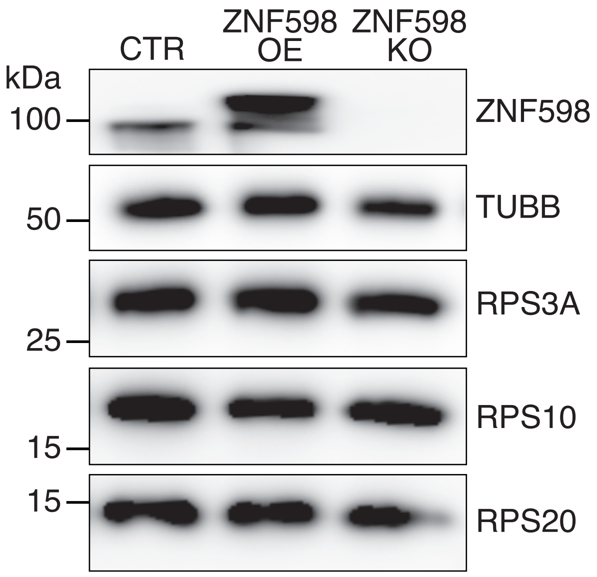

Supplementary Figure 14. Expression of the small ribosomal subunit proteins RPS3A, RPS10, and RPS20 is not affected by ZNF598.
Western blot analysis of expression of the indicated proteins in the CTR, ZNF598-OE, and ZNF598-KO cells. ZNF598 was detected by the anti-ZNF598 antibody (GeneTex).

Supplementary Figure 15

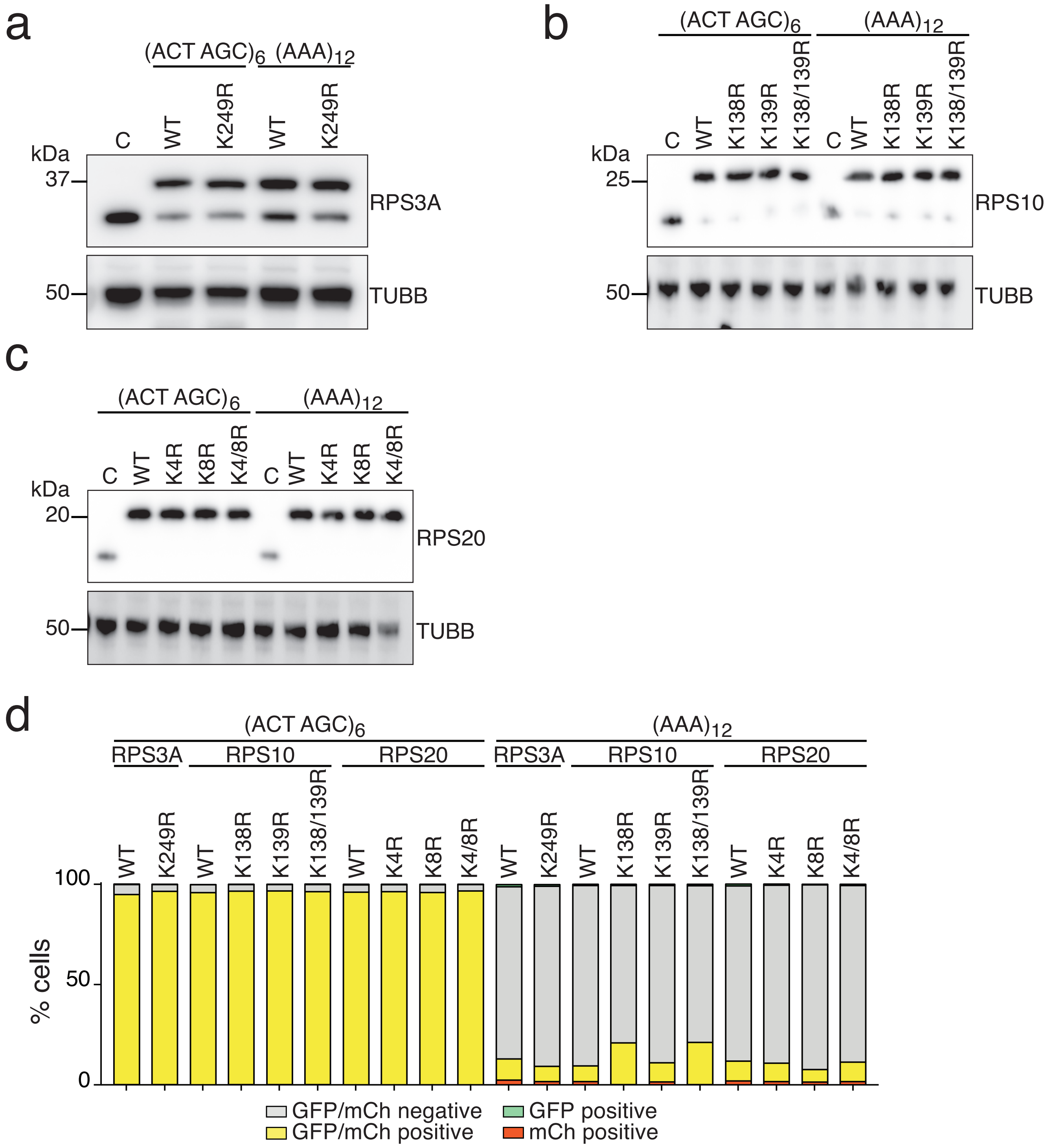

Supplementary Figure 15. Ubiquitination of lysine 138 of RPS10 is required for efficient RQC at premature polyA sequence.

(**a-c**) Western blot analysis of expression of the Myc-DDK-tagged wild-type or mutant ribosomal proteins RPS3A, RPS10, and RPS20 in HEK293 cells, constitutively expressing the indicated reporter. (**d**) Detection of GFP and mCherry fluorescent signals by FACS analyses in samples from (a-c), for reporter constructs containing (ACT AGC)6 and (AAA)12 linkers. Each experiment was performed in triplicate.

Supplementary Figure 16

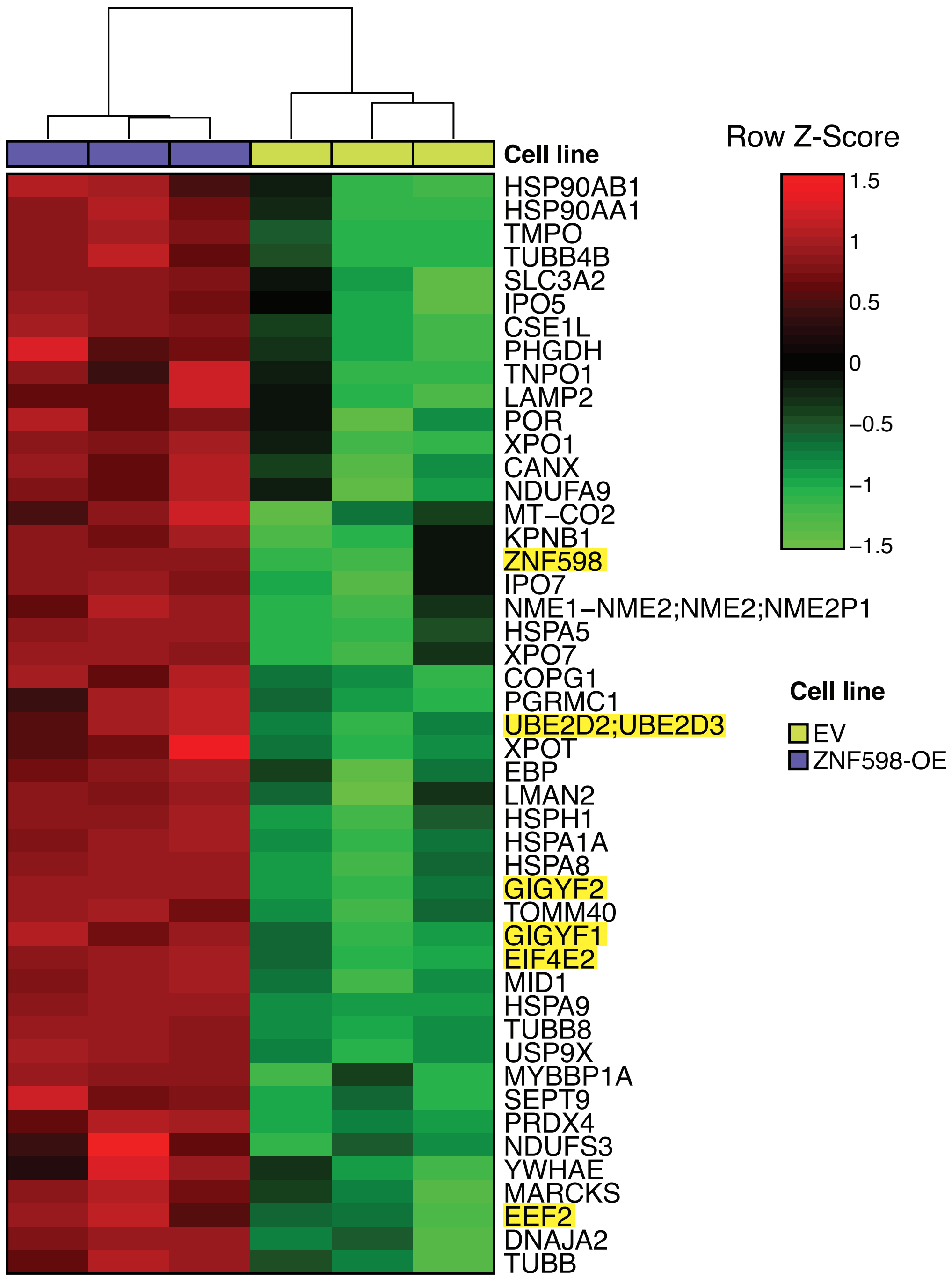

Supplementary Figure 16. Determination of ZNF598 interactome in HEK293 cells by immunoprecipitation and mass spectrometry (IP/MS) analysis.
Unsupervised hierarchical clustering using Euclidean distance and complete linkage for columns (samples) and rows (proteins) for 3xFlag-ZNF598-interacting proteins identified by anti-Flag antibody immunoprecipitation followed by mass spectrometry. HEK293 cells expressing 3xFlag peptide were used as control. Only proteins enriched in 3xFlag-ZNF598 samples and with FDR < 5% are shown. The color scale representing the z-score values is indicated. See also Figure 4b and Supplementary Data 6.

Supplementary Figure 17

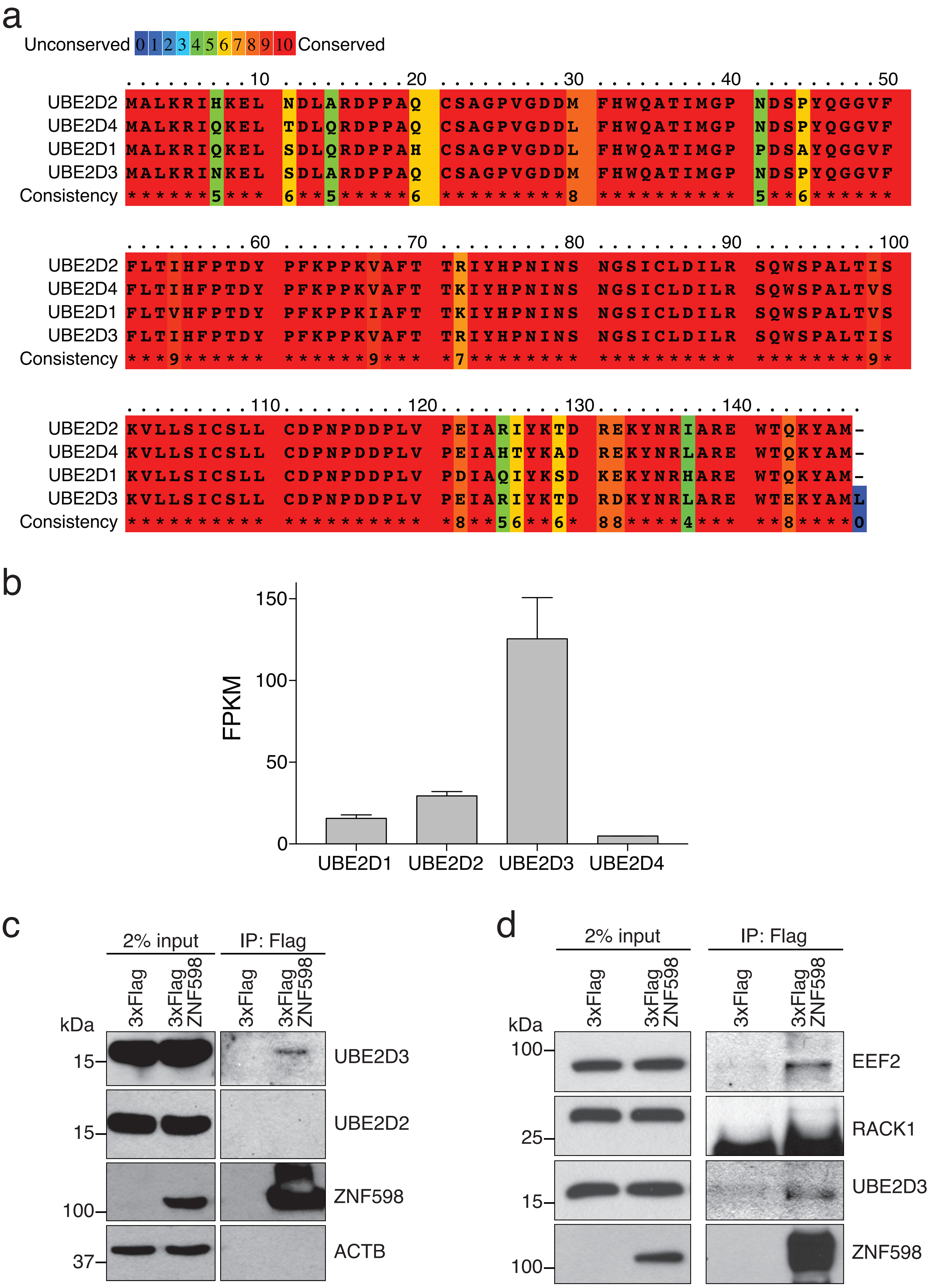

Supplementary Figure 17. ZNF598 interacted with eukaryotic translation elongation factor EEF2 and the UBE2D family of ubiquitin ligases.

(**a**) PRALINE alignment of the human UBE2D family members UBE2D1, UBE2D2, UBE2D3, and UBE2D4 proteins. Numbers and background color indicate the conservation scores. (**b**) polyA mRNA-Seq expression levels for UBE2D family members in HEK293 cells. (**c** and **d**) Co-immunoprecipitation with anti-Flag antibody in HEK293 cells expressing the 3xFlag or 3xFlag-ZNF598 proteins after RNase A treatment. Western blot probed for the indicated proteins. Flag/HA-ZNF598 was detected using primary anti-Flag antibody.

Supplementary Figure 18

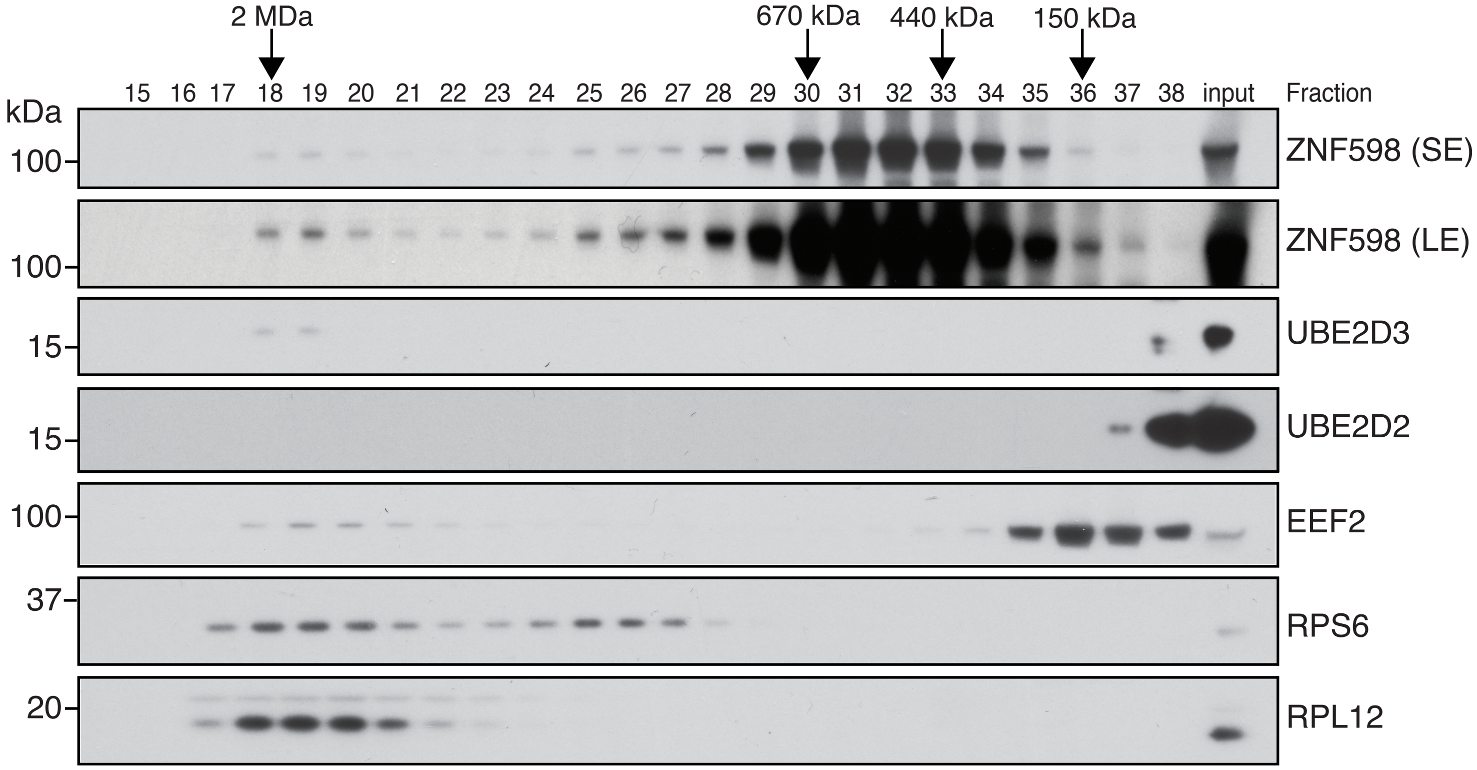

Supplementary Figure 18. Co-fractionation of EEF2 and UBE2D3 with ZNF598 in a >2 MDa complex. Size exclusion chromatography of ZNF598-OE cells. Fractions were analyzed by Western blotting for the presence of the indicated proteins. SE: Short exposure; LE: Long exposure. The elution position of the molecular mass markers is shown.

Supplementary Figure 19

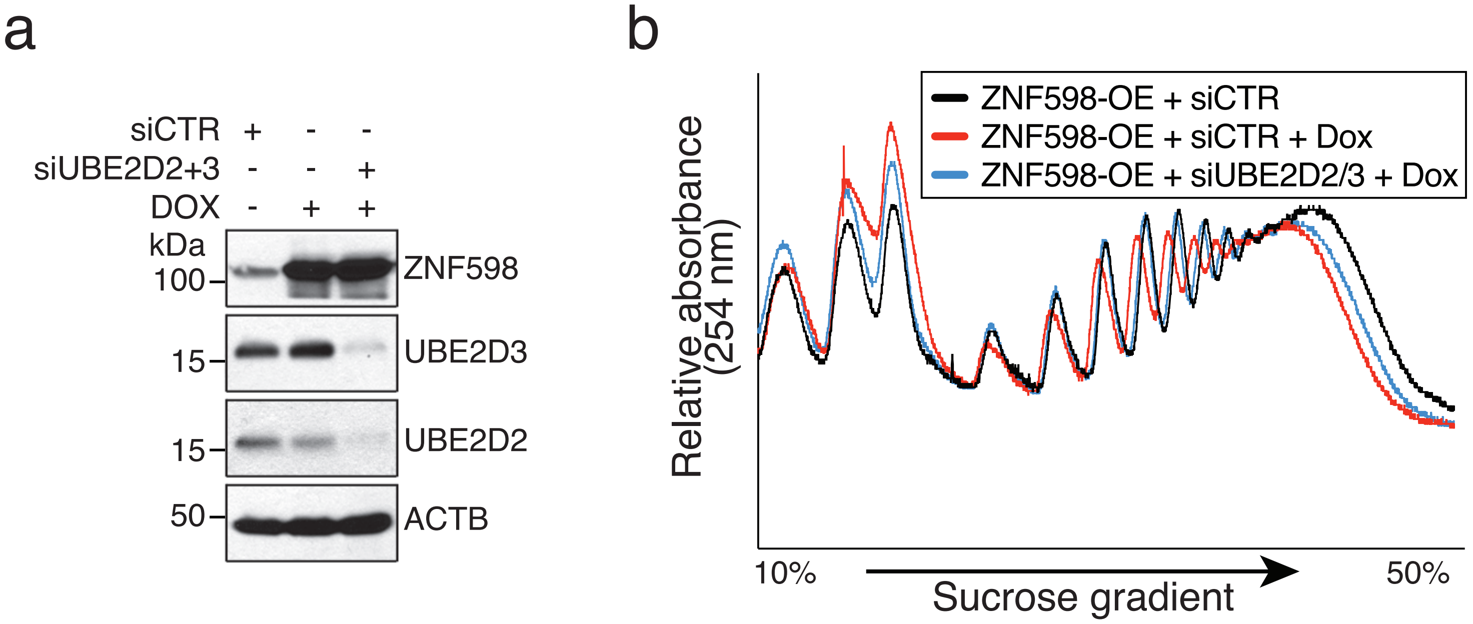

Supplementary Figure 19. siRNA-mediated depletion of UBE2D3 partially reverted the ZNF598-induced translation suppression.

(**a**) Western blot for the indicated proteins from ZNF598-OE cells following siRNA-mediated knockdown of UBE2D2 and UBE2D3. siCTR indicates the non-targeting control siRNA. (**b**) Polysome profiles of samples described in (a).

Supplementary Figure 20

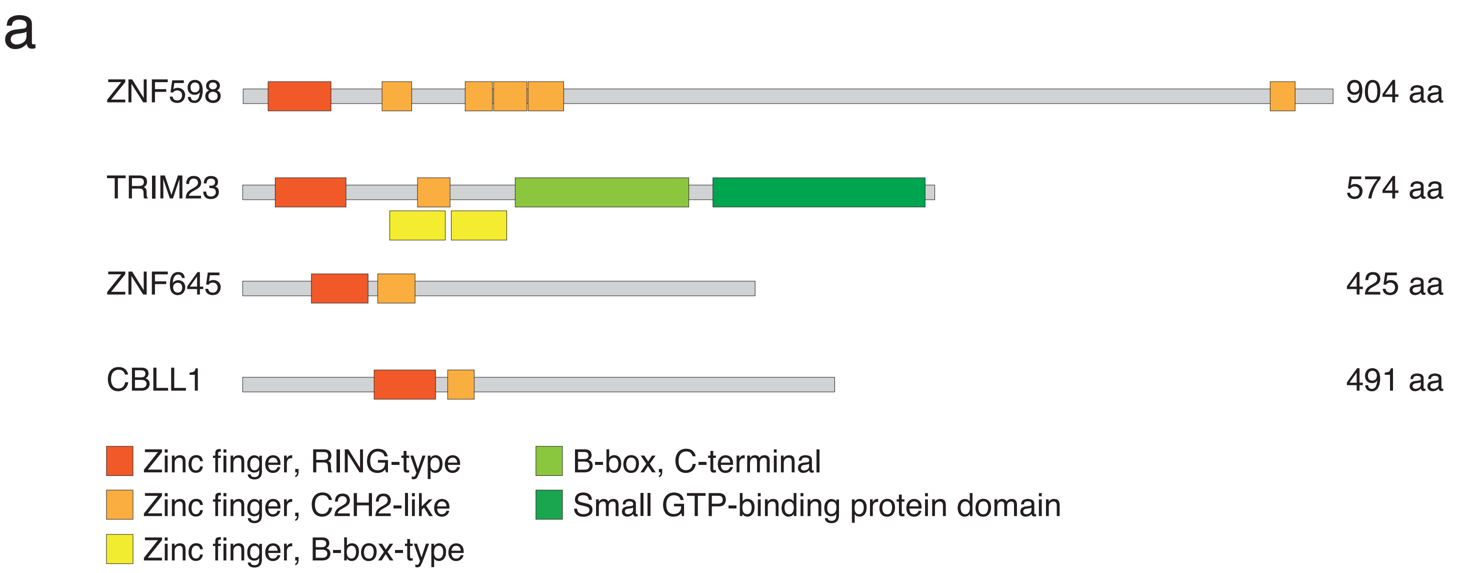

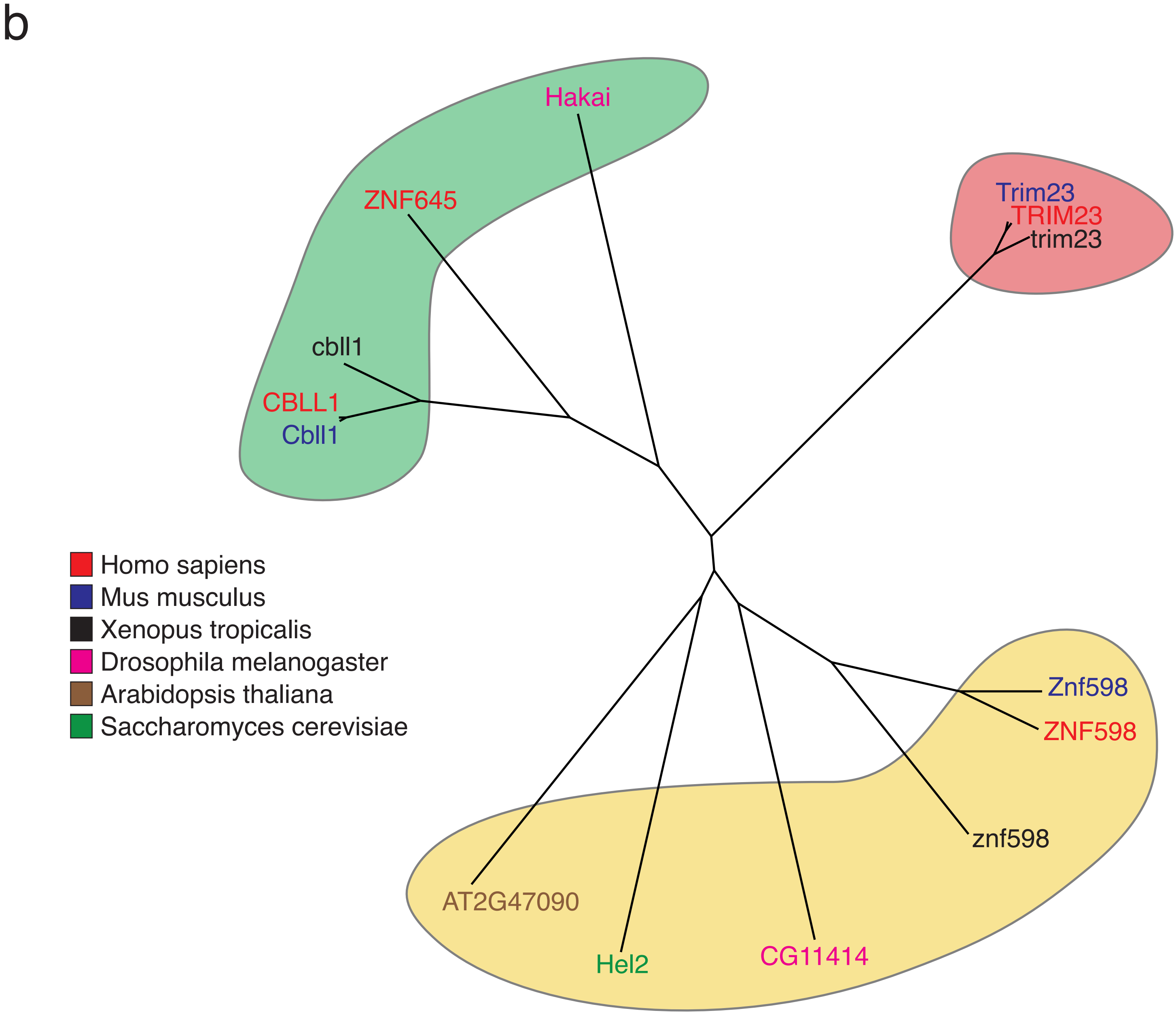

Supplementary Figure 20. The depth of evolutionary conservation of ZNF598 is unique in comparison to other RNA-binding protein with concurrent C2H2 and RING domains.
(**a**) Domain organization of the human ZNF598, TRIM23, ZNF645 and CBLL1 proteins was determined by Interpro. (**b**) Phylogenetic tree of ZNF598, TRIM23, ZNF645 and CBLL1 proteins in the indicated species.

Supplementary Figure 21

Figure 1

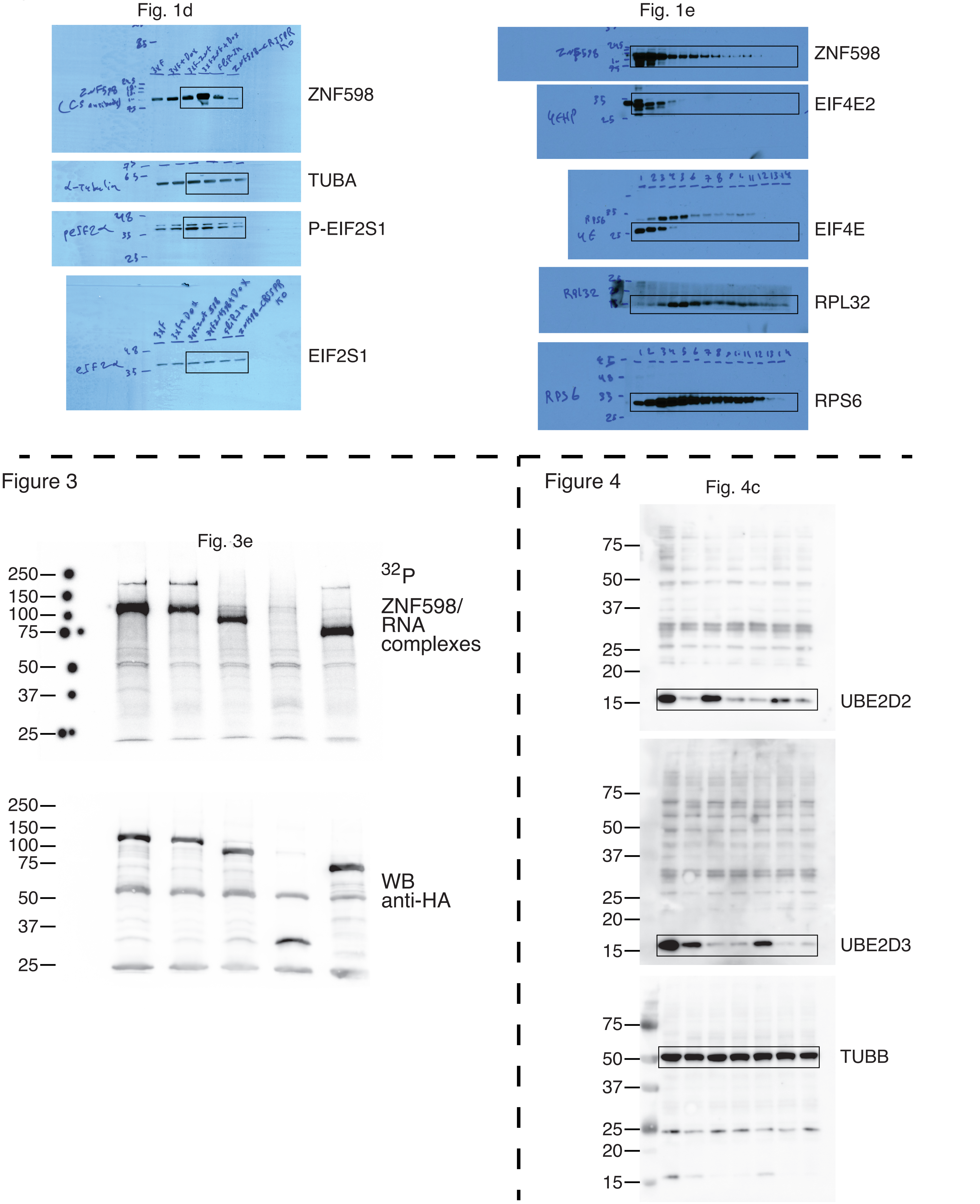

Supplementary Figure 21. Uncropped images of blots and gels used in main figures. Number of the corresponding figure is shown above the panels. Regions used for figures are boxed. Position of the molecular mass markers is shown on the left in kDa.

Supplementary Figure 22

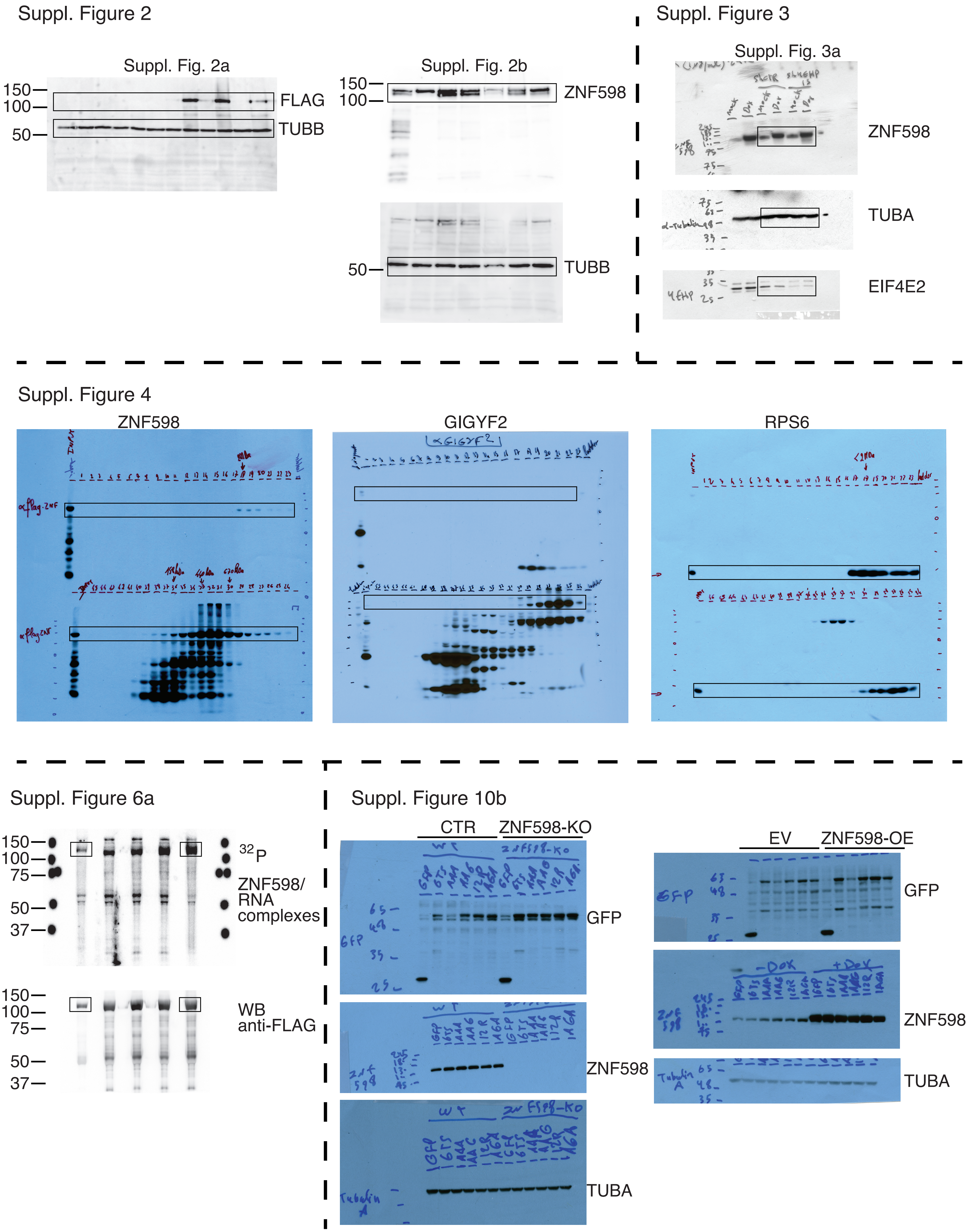

Suppl. Figure 12

Suppl. Fig. 12a

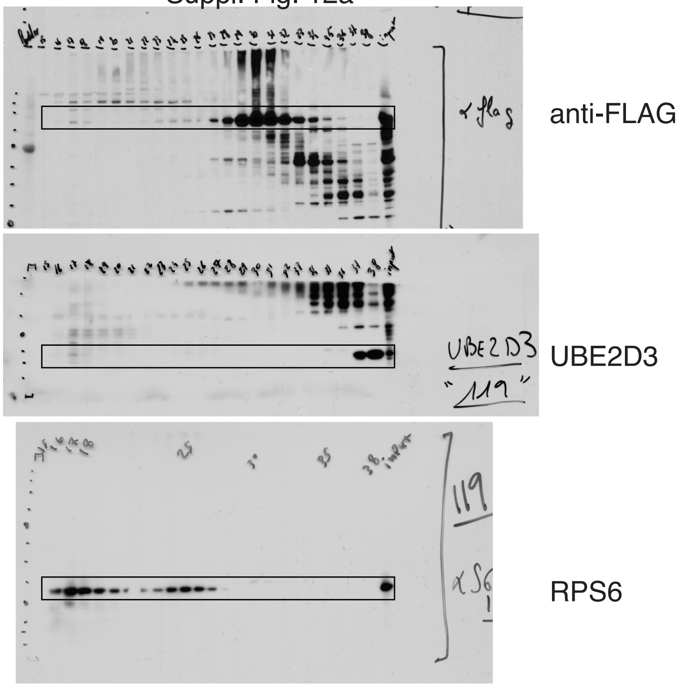

Suppl. Fig. 12b

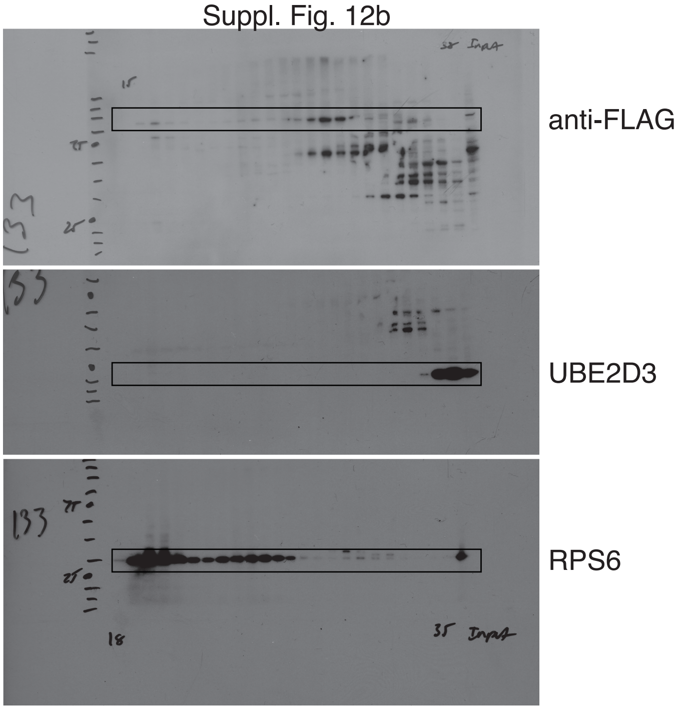

Suppl. Fig. 12c

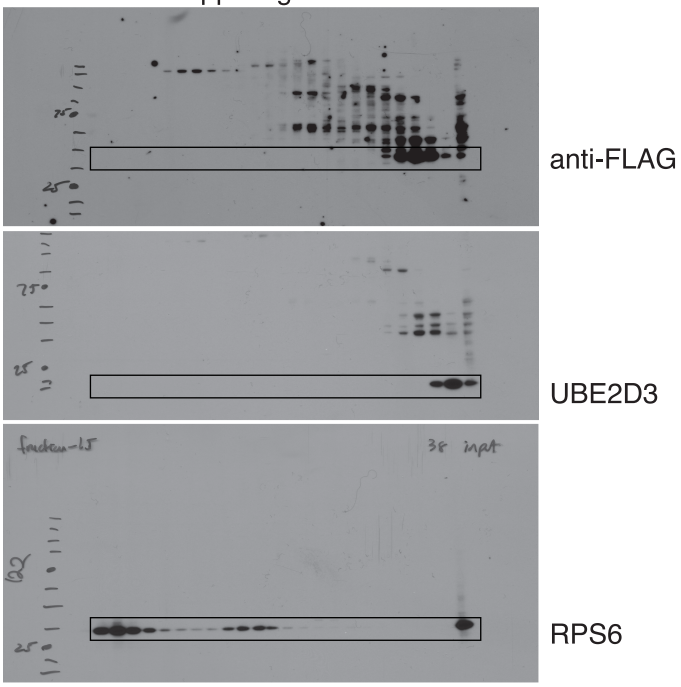

Suppl. Fig. 12d

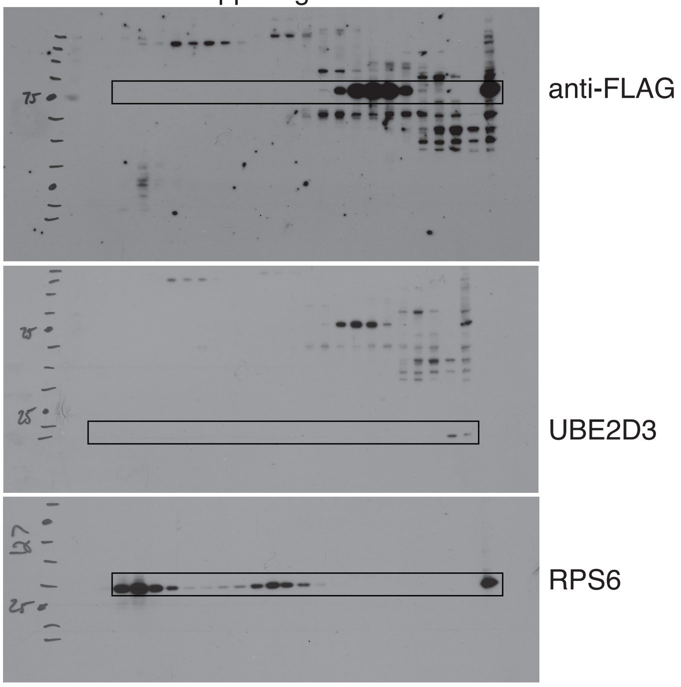

Suppl. Figure 14

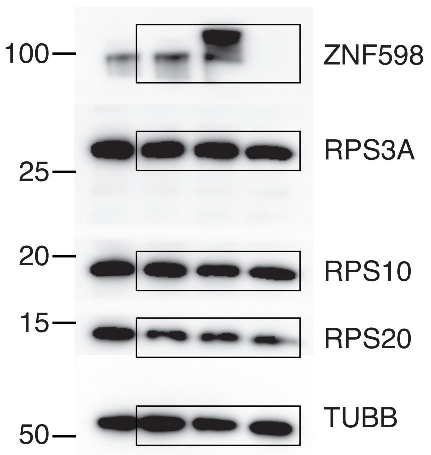

Suppl. Figure 15

Suppl. Fig. 15a

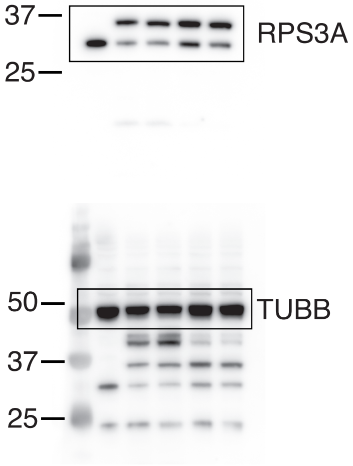

Suppl. Fig. 15b

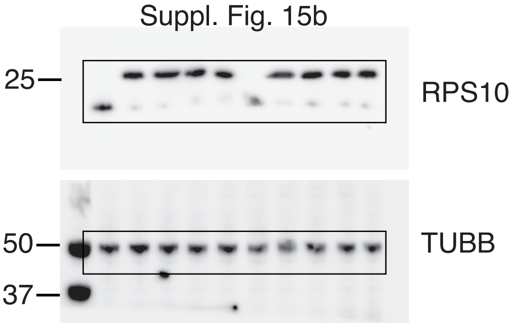

Suppl. Fig. 15c

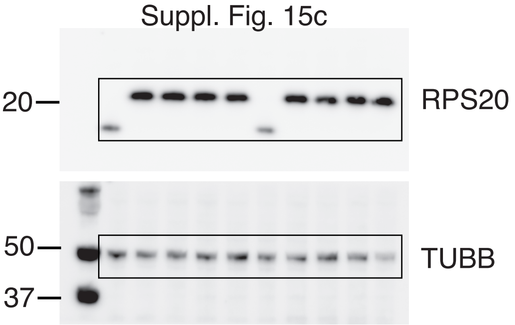

Suppl. Figure 17

Suppl. Fig. 17c

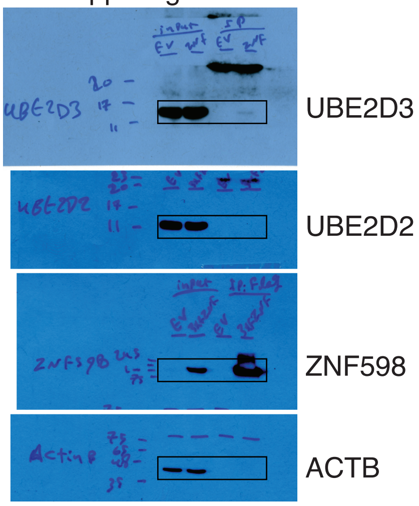

Suppl. Fig. 17d

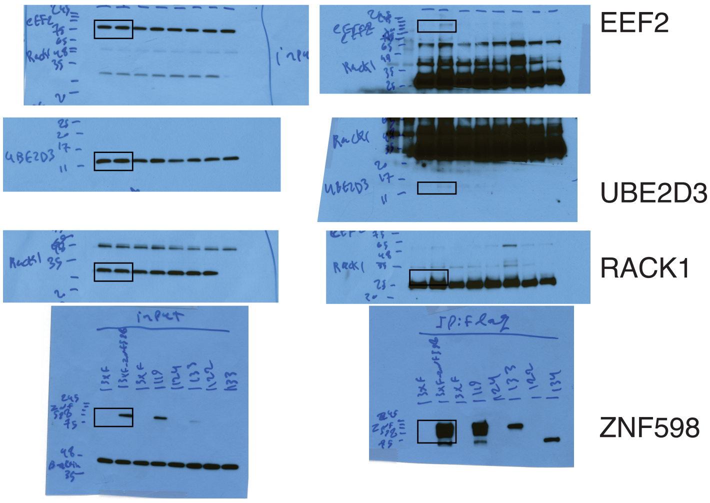

Suppl. Figure 18

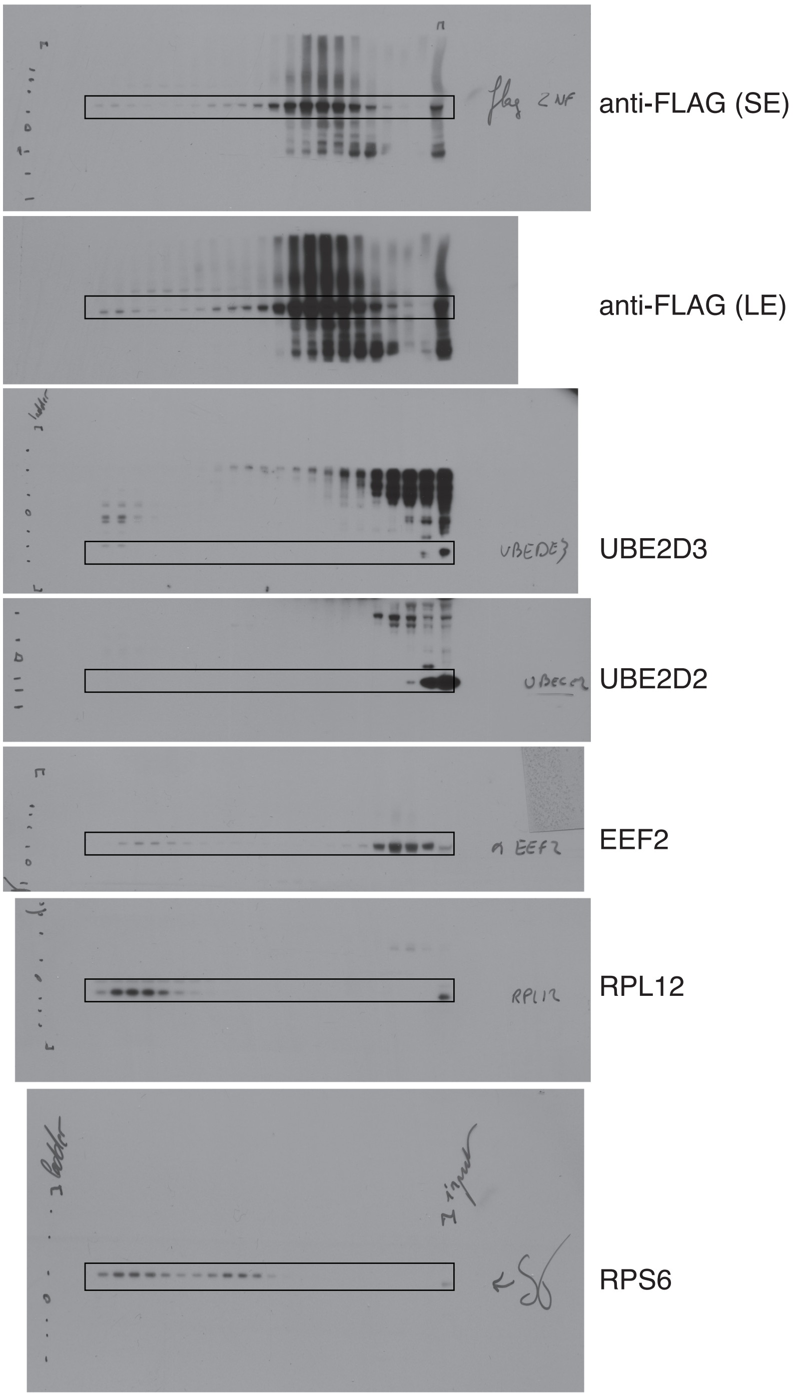

Suppl. Figure 19a

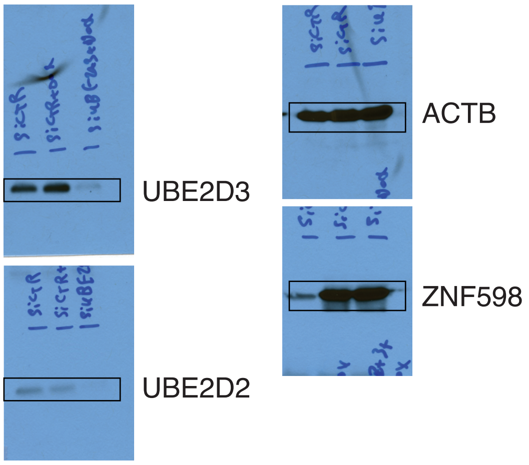

Supplementary Figure 22. Uncropped images of blots and gels used in supplemental figures. Number of the corresponding figure is shown above the panels. Regions used for figures are boxed. Position of the molecular mass markers is shown on the left in kDa.
